# Strong d–p orbital hybridization in cobalt porphyrin cages promotes electrochemical nitrate reduction to ammonia

**DOI:** 10.1039/d5sc07183f

**Published:** 2026-01-06

**Authors:** You Wu, Yangpeng Zhang, Hao Zhao, Yang Peng, Hailing Ma, Fangyuan Kang, Zhonghua Li, Yang Liu, Qichun Zhang

**Affiliations:** a School of Chemistry and Chemical Engineering, Harbin Institute of Technology Harbin 150001 P. R. China; b Department of Materials Science and Engineering, City University of Hong Kong Hong Kong SAR 999077 P. R. China; c School of Computer Science and Technology, Harbin Institute of Technology Harbin 150001 P. R. China; d Department of Chemistry, Center of Super-Diamond and Advanced Films (COSDAF), Hong Kong Institute of Clean Energy (HKICE), City University of Hong Kong Hong Kong SAR 999077 P. R. China

## Abstract

The electrocatalytic reduction of nitrate (NO_3_RR) to ammonia presents a viable solution for addressing nitrate pollution and offers an environmentally-friendly, energy-efficient alternative for industrial ammonia synthesis. However, the absence of efficient electrocatalysts impedes its industrial application. In this study, we constructed a porphyrin organic cage (PB-2) through the covalent-bonded self-assembly. Subsequently, metalized porphyrin organic cages, PB-M (M = Co, Ni, Cu), were synthesized *via* post-modification of PB-2. These PB-M catalysts were utilized to elucidate the reaction pathway and intrinsic structure–performance relationship of the NO_3_RR. Experimental results indicate that PB-Co exhibits the highest activity and ammonia selectivity (FE_NH3_ = 95.8 ± 1.06%, NH_3_ yield rate = 995.5 ± 28.4 µmol h^−1^ mg_cat_^−1^). Theoretical calculations reveal that the d–p orbital hybridization between the Co 3d orbital in PB-Co and the NO_3_^−^ 2p orbital is the strongest one. PB-Co possesses a high d-band center of −0.97 eV and high adsorption energies for NO_3_^−^ and H_2_O, promoting charge transfer and the production of active hydrogen, thereby reducing the activation energy barrier of NO_3_^−^. This research illuminates the intrinsic structure–activity relationship of metalized PB-M for the NO_3_RR, potentially providing valuable insights for the design of efficient electrocatalysts.

## Abstract 2

Three metalized porphyrin organic cages, PB-M (M = Co, Ni, Cu), were synthesized. Due to the more positive d-band center and stronger d–p hybridization, the highest faradaic efficiency and ammonia yield were achieved by PB-Co.

## Graphical abstract

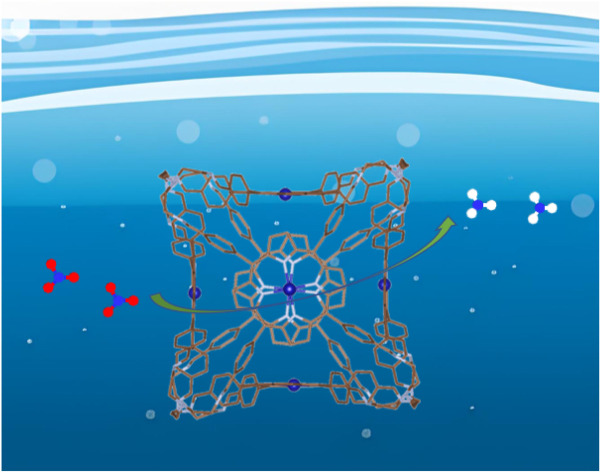

## Introduction

Ammonia serves as a fundamental component in contemporary industry and agriculture, primarily owing to its pivotal role in fertiliser production, which is indispensable for global food production.^1–3^ Prior to the advent of the 20th century, manure constituted the primary source of nitrogen fertilizer required for agricultural production, thereby posing a severe constraint on food production.^4^ The emergence of the Haber–Bosch process made large-scale industrial synthesis of ammonia a reality, markedly increasing food production and protecting billions of people from hunger.^5–7^ Nevertheless, this process is characterized by high energy intensity, profound dependence on fossil fuels, and significant greenhouse gas emissions.^8–10^ Consequently, the development of a clean and sustainable technology for NH_3_ synthesis becomes imperative. In the past few years, electrochemical N_2_ reduction to ammonia (NRR) has received increasing attention.^11–14^ However, utilizing N_2_ as a nitrogen source presents several challenges, including its high NE002N dissociation energy, limited solubility, and the potentially competing hydrogen evolution reaction, resulting in the much lower efficiency of the NRR comparing to commercial requirements.^15–19^ The development of industry and agriculture has led to a significant increase in the generation of nitrate pollutants. These contaminants, often inadequately treated, permeate groundwater and surface water, posing grave threats to both human health and ecosystem equilibrium.^20,21^

The electrocatalytic reduction of nitrate (NO_3_RR) provides an environmentally friendly, energy-saving, and potentially scalable solution to address nitrate pollution. The NO_3_RR employs renewable and clean electrical energy to drive the reduction reaction, thereby effectively decoupling ammonia synthesis from fossil fuels. This innovative technology not only reduces greenhouse gas emissions but also addresses the critical challenge of nitrate contamination in water bodies.^22–24^ Due to the complexity of the NO_3_RR process and intermediate products, the development of highly efficient and selective electrocatalysts poses a significant challenge.^25–27^ Efficient NO_3_RR requires highly active, selective, and stable electrocatalysts. Porphyrin serves as the active center in numerous biological enzymes and acts as a significant biomimetic catalyst, possessing high catalytic efficiency and selectivity during the catalytic reaction process.^28,29^ The utilization of porphyrin molecules in the NO_3_RR has been extensively documented over the years.^30–32^ However, the low density of active sites and the tendency to aggregate have hindered the practical application of porphyrin molecule catalysts. The integration of porphyrins into porous materials, such as covalent organic frameworks (COFs), metal–organic frameworks (MOFs) and porous organic cages (POCs) has become a trend.^33–38^ POCs are an emerging sub-class of crystalline porous materials that have garnered significant attention in recent years due to their exceptional properties. In 2015, Kim *et al.* synthesized two porphyrin boxes (PB-1 and PB-2) through dynamic imine condensation reactions.^39^ These two porphyrin boxes are covalent organic cages with a rhombic cuboctahedral geometry, constructed from six porphyrin units and eight triamine linkers. The hollow characteristics of octahedral porphyrin boxes and the excellent chemical properties of porphyrin units have enabled Kim *et al.* to apply them in the fields such as electrocatalysis and photocatalysis.^40–43^ Due to the open intrinsic and inter-cage pores of porphyrin organic cages, when metal ions coordinate with porphyrin, small molecules can readily diffuse to the metal ion sites, thereby facilitating the occurrence of catalytic reactions. The porphyrin organic cages have the structural characteristics of enzyme catalysts, providing valuable insights for the study of supramolecular catalysts and potential avenues for the research of biomimetic catalysts.

In this study, we successfully synthesized a porphyrin porous organic cage (PB-2), which can be utilized as a support for Single Metal Atoms (SACs). The incorporation of metal atoms into PB-2 significantly enhances its electrocatalytic activity for the NO_3_RR. Specifically, PB-Co demonstrates excellent electrocatalysis efficiency for the NO_3_RR with a FE_NH3_ of 95.8 ± 1.06% and a corresponding NH_3_ yield of 995.5 ± 28.4 µmol h^−1^ mg_cat_^−1^. The d-band center of PB-Co is −0.97 eV, which is closer to the Fermi level. DFT analysis shows that the d–p orbital hybridization between the Co 3d orbital in PB-Co and the NO_3_^−^ 2p orbital is the most robust. The experimental and theoretical results also suggest that PB-Co adsorbs H_2_O more easily and produces active hydrogen (*H). Furthermore, PB-Co exhibits the lowest reaction barrier, thereby facilitating the reduction of NO_3_^−^.

## Results and discussion

**Scheme 1:**
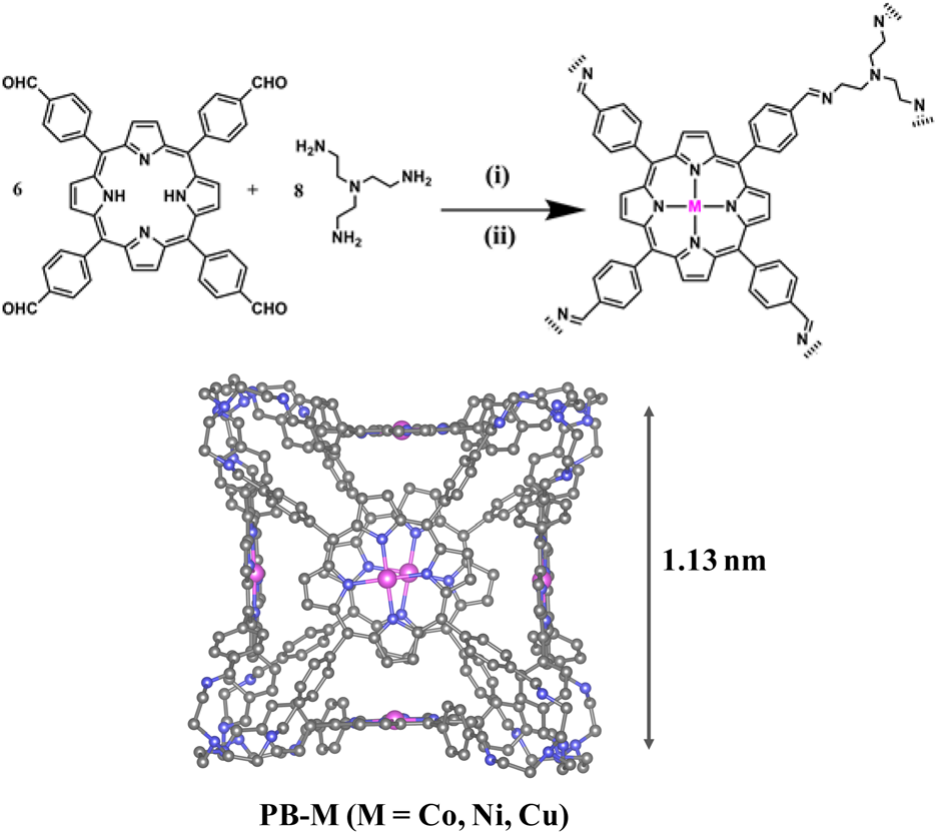
Synthesis of PB-2 and PB-M (M = Co, Ni, Cu). (i) TFA, *o*-dichlorohenzene, 80 °C, 5d; (ii) THF, Co^2+^/Ni^2+^/Cu^2+^ salt, 70 °C, 24 h.

**Fig. 1:**
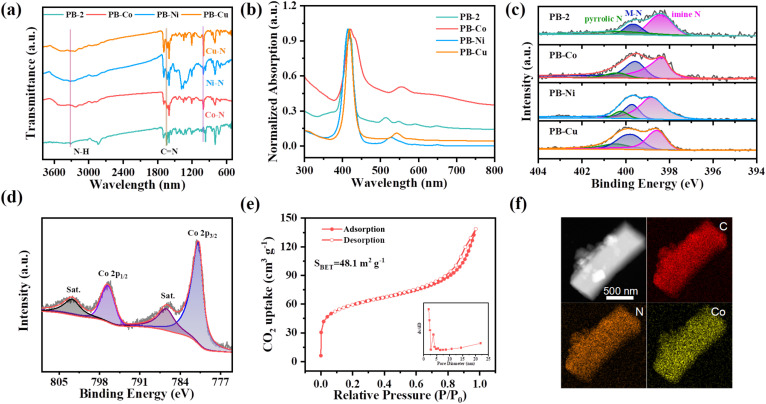
(a) FT-IR spectra of PB-2, PB-Co, PB-Ni and PB-Cu. (b) UV-vis absorption spectra of PB-2, PB-Co, PB-Ni and PB-C. (c) High-resolution N 1s XPS spectra of PB-2, PB-Co, PB-Ni and PB-Cu. (d) High-resolution Co 2p XPS spectra of PB-Co. (e) CO_2_ adsorption–desorption curves of PB-Co. (f) TEM mapping image of PB-Co.

The cage used in our work was PB-2, first reported by the Kim group.^39^ PB-2 was synthesized through dynamic imine condensation between *meso*-tetrakis(4-formylphenyl)porphyrin (*p*-Por-CHO) and tris(2-aminoethyl)amine (TREN), achieving a [6 + 8] architecture as designed (Scheme 1). The successful synthesis of PB-2 was confirmed by MALDI-TOF and ^1^H NMR spectroscopy. The mass spectral profile revealed a prominent molecular ion peak at *m*/*z* 5097.3, correlating with the proposed six-porphyrin/eight-TREN stoichiometry (Fig. S1). ^1^H NMR spectroscopy showed that the imine proton signal appeared at around 8.44 ppm (H_e_–C=N) for PB-2 (Fig. S2).^44^ Then, the metal atom (Co, Ni, and Cu) was incorporated into PB-2 by refluxing the mixture of PB-2 and corresponding metal salts. The successful metallization of PB-2 in the presence of different metal salts was verified using MALDI-TOF mass spectrometry (Fig. S1). Powder X-ray diffraction was employed to characterize the crystal structure of PB-2 and PB-M (Fig. S3). The similar characteristic peaks of PB-2 and PB-M were observed, indicating that the crystal structure of PB-2 was not destroyed by chelating metals. Fourier transform infrared (FT-IR) spectroscopy of PB-2 and PB-M (M = Co, Ni, Cu) was also studied (Fig. 1a). Specifically, the peak at 1640 cm^−1^ can be assigned to the C=N bond, the disappeared peak at 3332 cm^−1^ corresponds to the stretching of N–H on the porphyrin ring and the newly appeared peak at 1000 cm^−1^ (M–N–C) confirm the successful metallization of PB-2.^45^ The UV-vis spectra of PB-2 displayed a strong Soret band around 420 nm and four Q bands between 500 and 670 nm (Fig. 1b). The Soret band arises from the a_1u_(π) → e_g_(π*) transition, whereas the Q bands correspond to a_2u_(π) → e_g_(π*) transition, respectively. The reduction in the number of Q bands in PB-Co, PB-Ni, and PB-Cu is attributed to the increased symmetry of the porphyrin upon coordination of metal ions, indicating the coordination of metal ions within PB-2.^46^

X-ray photoelectron spectroscopy (XPS) tests were conducted to investigate the surface chemical states as well as the elemental composition of these samples. The C 1s XPS spectrum of PB-2 revealed four distinct peaks at 284.8, 285.6, 286.7 eV, and 289.4 eV, which can be attributed to C–C, C–N, C=O, and N–C=O bonds, respectively (Fig. S4). In the N 1s XPS spectra of PB-M (Fig. 1c), three distinct peaks were observed, attributable to imine nitrogen, metal-coordinated nitrogen and pyrrolic N, respectively.^47^ PB-Co exhibited characteristic Co^2+^ signatures in its 2p spectrum with satellite peaks (Fig. 1d). PB-Ni showed analogous divalent characteristics (Fig. S5). In the case of PB-Cu, peaks for both divalent and monovalent copper were observed, and the ratio of Cu^+^/(Cu^2+^+Cu^+^) in the PB-Cu samples is 30.9%.^48^ The loading amount of metal atoms was determined by inductively coupled plasma optical emission spectrometry (ICP-OES) analysis, with Co, Ni, and Cu contents of 6.04%, 6.27%, and 7.18%, respectively (Table S1).

Due to the relatively weak adsorption capacity of cages for N_2_ at 77 K, the porosity of cage is mostly estimated by CO_2_ adsorption at 195 K, which is common in porous molecular materials. As shown in Fig. 1e and S6, all of these samples exhibited typical type IV sorption isotherm curves, a characteristic evidence for mesoporous structures. The Brunauer–Emmett–Teller (BET) surface areas were calculated to be 242.8 m^2^ g^−1^, 48.1 m^2^ g^−1^, 51.6 m^2^ g^−1^, and 59.4 m^2^ g^−1^ for PB-2, PB-Co, PB-Ni, and PB-Cu, respectively. The pore-size distributions of PB-M showed a main peak centered at around 3.4 nm. The microstructure of these samples was characterized using Scanning Electron Microscopy (SEM) and Transmission Electron Microscopy (TEM). SEM (Fig. S7) and TEM (Fig. S8) images revealed that PB-2 and PB-M (M = Co, Ni, Cu) have similar morphological characteristics. The element mapping image shows the uniform distribution of Co, C, and N in PB-Co (Fig. 1f). Additionally, the morphology and element distribution of PB-2, PB-Ni, and PB-Cu are displayed in Fig. S9–S11, respectively.

**Fig. 2:**
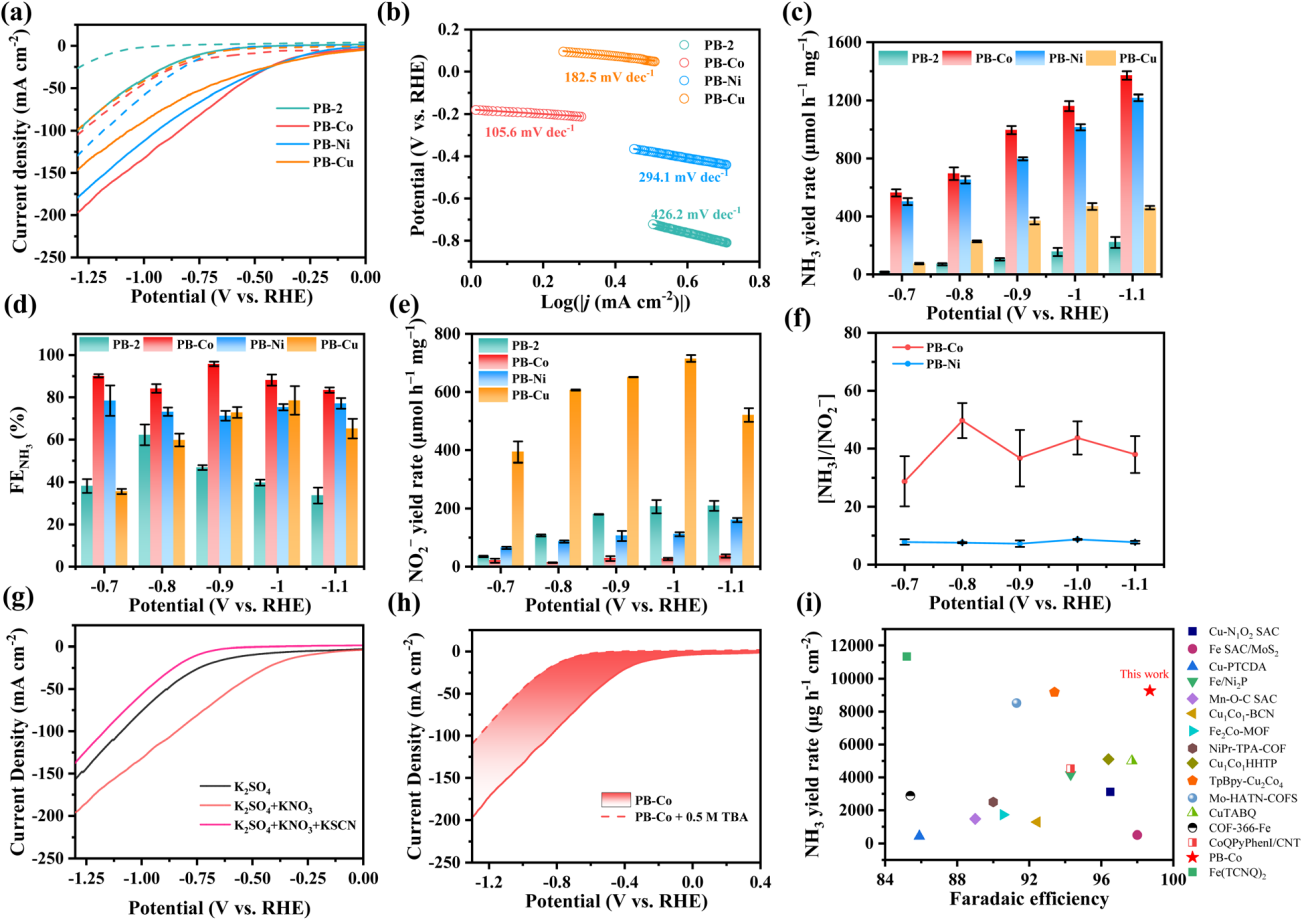
(a) LSV curves of PB-2 and PB-M in Ar-saturated 0.5 M K_2_SO_4_ (dashed line) and 0.5 M K_2_SO_4_ + 0.1 M KNO_3_ (solid line) electrolyte. (b) The Tafel plots of PB-2 and PB-M in 0.5 M K_2_SO_4_ + 0.1 M KNO_3_. (c) NH_3_ yield rate of PB-2 and PB-M at different potentials. (d) FE_NH3_ of PB-2 and PB-M at different potentials. (e) NO_2_^−^ yield rate of PB-2 and PB-M at different potentials. (f) [NH_3_]/[NO_2_^−^] at different potentials. (g) LSV curves of PB-Co in 0.5 M K_2_SO_4_, 0.5 M K_2_SO_4_ + 0.1 M KNO_3_, and 0.5 M K_2_SO_4_ + 0.1 M KNO_3_ + 0.1 M KSCN electrolytes. (h) Comparison of current density change by adding 0.5 M TBA into KNO_3_-containing electrolyte. (i) Comparison of NO_3_RR performance of PB-Co with other electrocatalysts.

In order to evaluate the electrochemical performance of PB-2 and PB-M (M = Co, Ni, Cu), these samples were dropcast onto a 1 × 1.5 cm^2^ carbon paper (CP) with a mass loading of 0.5 mg cm^−2^ and tested in a H-type electrolytic cell with a three-electrode system. Fig. 2a depicts the linear sweep voltammetry (LSV) curves of the samples in Ar–saturated 0.5 M K_2_SO_4_ with and without 0.1 M KNO_3_.

In 0.5 M K_2_SO_4_ electrolyte, only the hydrogen evolution reaction (HER) occurs on the catalyst surface, and the current density of the PB-Co catalyst does not significantly increase until the voltage is lower than −0.25 V (*vs.* RHE, the same hereinafter). The addition of KNO_3_ significantly increased the current density of PB-2 and PB-M (M = Co, Ni, Cu) in the electrolyte. For PB-Co catalysts, the current density starts to take off at −0.25 V and increases to 200 mA cm^−2^ at −1.35 V. In contrast, the maximum current density of the PB-2 catalyst within the test voltage range is less than 100 mA cm^−2^, which is the proof of the great NO_3_RR activity for PB-Co. As illustrated in Fig. 2b, the Tafel slope of PB-Co (105.6 mV dec^−1^) is notably smaller than those of PB-2 (426.2 mV dec^−1^), PB-Ni (294.1 mV dec^−1^), and PB-Cu (182.5 mV dec^−1^). This indicates that PB-Co exhibits the fastest reaction kinetics. The analysis results indicate that both PB and PB-M catalysts have potential ability toward the NO_3_RR. Compared with the PB-2 catalyst, all metalized PB-2 catalysts showed enhanced catalytic performance.

Quantification of NH_3_ yields was performed by UV-vis spectrophotometry using the indophenol blue method. The NH_3_ yield and corresponding faradaic efficiency (FE) at different potentials are calculated and shown in Fig. 2c and d. Notably, PB-Co exhibited the highest FE toward NH_3_ within the −0.70 to −1.10 V range, achieving a peak faradaic efficiency (95.8 ± 1.06%) at −0.9 V, with a corresponding NH_3_ yield of 995.5 ± 28.4 µmol h^−1^ mg_cat_^−1^. We compared the NH_3_ yield rates between molecular cobalt porphyrin (Co-TPP) and PB-Co, found that the NH_3_ yield rate of PB-Co was approximately 4 times higher than that of Co-TPP (Fig. S14). Therefore, integrating Co into a porpyhrin organic cage can effectively enhance the catalytic performance. Interestingly, although the PB-Cu catalyst shows a relatively high current density in the LSV curve, it exhibited the lowest faraday efficiency and NH_3_ yield among the PB-M catalysts. Therefore, we calculated the yield rate and FE of NO_2_^−^, a significant byproduct of nitrate reduction (Fig. 2e and S15). The PB-Cu catalyst showed the highest yield rate of NO_2_^−^. At −0.7 V, the yield rate and FE of NO_2_^−^ were 393.8 ± 36.3 µmol h^−1^ mg_cat_^−1^ and 11.8 ± 0.07%, respectively. As the potential decreased, the FE of NO_2_^−^ also showed a downward trend. In order to visually display the dominant product after the reaction, the yield rate ratio of NH_3_ to NO_2_^−^ in the product was defined as [NH_3_]/[NO_2_^−^]. As shown in Fig. 2f and S15, for the PB-2 catalyst, the values of [NH_3_]/[NO_2_^−^] were less than 1, indicating that NO_2_^−^ is the main product, with low selectivity for NH_3_. As for PB-Co and PB-Ni, the values of [NH_3_]/[NO_2_^−^] were much greater than 1.0, indicating high selectivity for NH_3_. As for PB-Cu, within the test potential range, the values of [NH_3_]/[NO_2_^−^] were much smaller than 1.0, indicating that NO_2_^−^ is the main product. The reason for this phenomenon is that *NO_2_^−^ is easy to desorb on PB-Cu. The theoretical calculation show that the desorption free energy of NO_2_^−^ on PB-Cu is a negative value, indicating that the desorption of NO_2_^−^ on PB-Cu is a spontaneous process, resulting in the selectivity of ammonia synthesis on PB-Cu being lower than that of PB-Ni and PB-Co (Fig. S16).^49^

To elucidate the primary active centers and mechanism of PB-M in the NO_3_RR, systematic KSCN poisoning experiments were implemented. This approach leverages the strong affinity of SCN^−^ for metal sites, thereby blocking nitrate adsorption through competitive binding.^50,51^ When introduced into the combined electrolyte system (0.5 M K_2_SO_4_ + 0.1 M KNO_3_), the KSCN additive induced a marked decrease in current densities compared to the KSCN-free control, as evidenced by electrochemical characterization (Fig. 2g and S17). Notably, the suppressed current levels fell below those observed under nitrate-free conditions, confirming the predominant role of metal centers in PB-M (M = Co, Ni, Cu) for the NO_3_RR. The NO_3_RR is a process involving the transfer of nine protons, so active hydrogen (*H) plays an important role in the NO_3_RR. In order to study the ability of different metals to produce *H, 0.5 M tert-butanol (TBA) was added to the electrolyte, where TBA acts as a scavenger for *H. The integral area of current density change after the addition of TBA for PB-Co (68.38) is larger than that of PB-Ni (42.64) and PB-Cu (19.51) (Fig. 2h and S18), indicating that PB-Co can produce more *H. We compared the performance of PB-Co with other recently reported electrocatalysts for the NO_3_RR, and the NH_3_ yield rate and FE_NH3_ of PB-Co exceed those of the majority of catalysts reported in previous literature (Fig. 2i and Table S2).

**Fig. 3:**
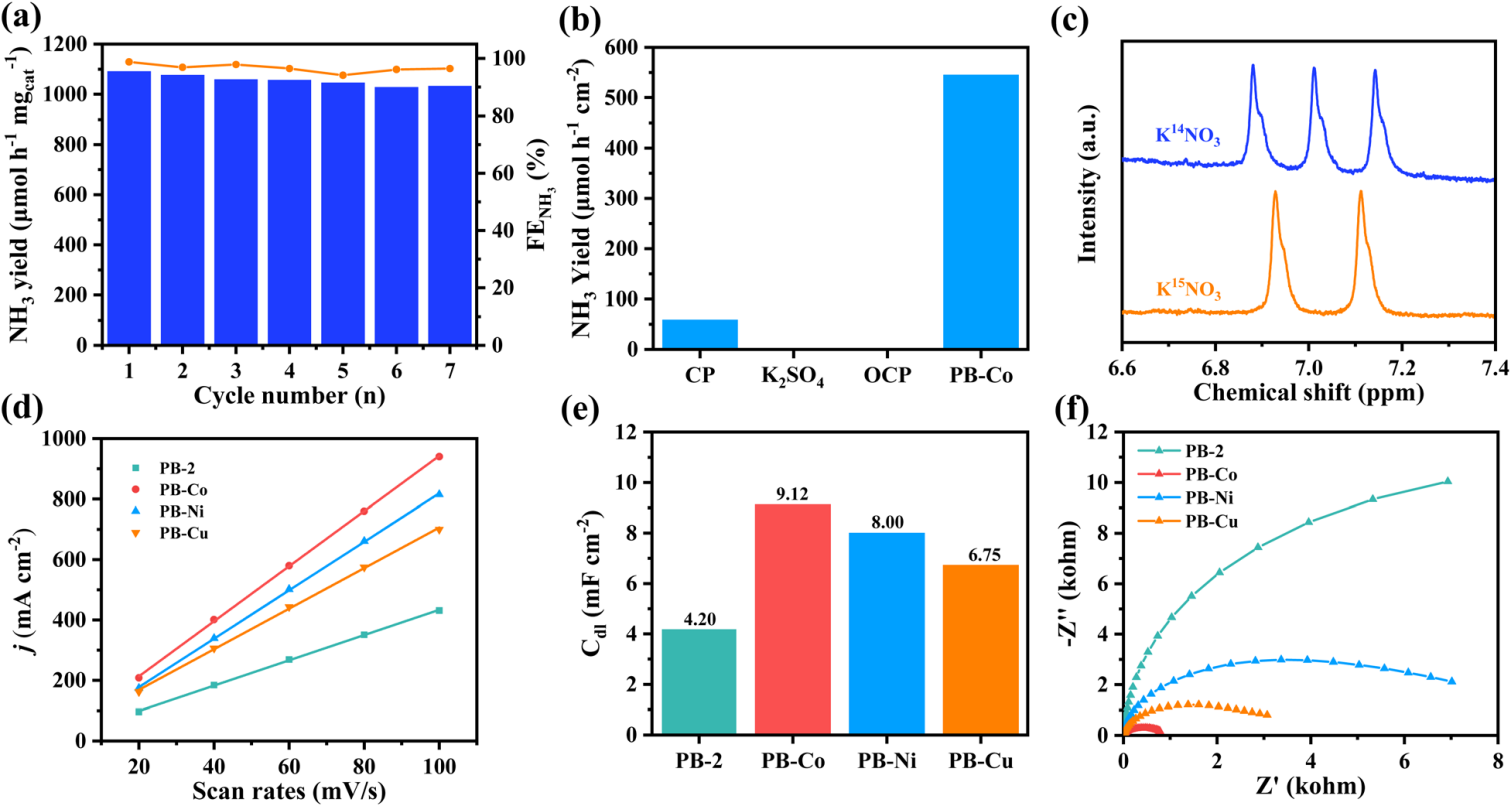
(a) The cycling tests of PB-Co at −0.90 V *vs.* RHE. (b) NH_3_ yield of PB-Co under different test conditions. (c) ^1^H NMR spectra of the electrolyte used in the NO_3_RR with K^15^NO_3_ or K^14^NO_3_ as nitrogen sources. (d) Current density at different scanning rates. (e) Double layer capacitance of PB-2 and PB-M. (f) Nyquist plots of PB-2 and PB-M.

The stability of catalysts is a crucial factor in assessing their potential for industrial applications. Seven cycles of electrolysis with a PB-Co electrode were carried out at −0.9 V, each cycle lasting 1 hour (Fig. 3a). It is clear that the FE and yield rate for NH_3_ slightly decreased, indicating excellent catalytic stability of PB-Co. Post-electrolysis characterization of PB-Co through FT-IR (Fig. S20), XPS (Fig. S21), and SEM (Fig. S22) analyses demonstrated minor structural alterations, conclusively verifying the exceptional stability of the catalyst under operational conditions. MALDI-TOF analysis also demonstrated that the molecular structure of PB-Co remained intact (Fig. S23). ICP-MS analysis of the electrolyte (after electrocatalysis for 4 h) indicated that the leached Co, Ni, and Cu concentrations were below the detectable limit (0.1 µg L^−1^). Therefore, PB-M has great stability during the electrolysis. To corroborate the source of produced ammonia, the NO_3_RR performance of carbon paper (CP) was evaluated by the NH_3_ yield (Fig. 3b). As expected, CP exhibits low NO_3_RR activity. PB-Co was used as the working electrode in 0.1 M K_2_SO_4_ electrolyte, no NH_3_ production was detected. Similarly, with PB-Co as the working electrode and 0.5 M K_2_SO_4_ + 0.1 M KNO_3_ as the electrolyte, no ammonia was detected in the electrolyte under open circuit voltage (OCP). Isotope labeling experiments using K^14^NO_3_ or K^15^NO_3_ as a nitrogen source, followed by product identification *via*^1^H-NMR, were performed. As shown in Fig. 3c, three peaks are observed in the ^1^H-NMR spectrum after 2 h of electrolysis when using K^14^NO_3_ as the electrolyte, corresponding to ^14^NH_4_^+^. Only double peaks corresponding to ^15^NH_4_^+^ are identified in the ^1^H-NMR spectrum when using K^15^NO_3_ as the electrolyte, suggesting that the produced ammonia totally originates from the electrochemical NO_3_RR.

To evaluate the true catalytic performance of samples, it is necessary to obtain electrochemically active surface areas (ECSAs); however, we cannot directly calculate the ECSAs. Due to the relationship between double-layer capacitance (*C*_dl_) and ECSA, we can roughly calculate the ECSA by calculating the *C*_dl_.^52,53^ Cyclic voltammetry measurements were conducted (Fig. S24), and by fitting the current density Δ*j* at different scanning rates (20 to 100 mV s^−1^), the *C*_dl_ values of PB-2 and PB-M were obtained (Fig. 3d and e). The results revealed that the *C*_dl_ value for PB-Co is 9.12 mF cm^−2^, which is notably higher than that for PB-Ni (8.02 mF cm^−2^), PB-Cu (6.72 mF cm^−2^), and PB-2 (4.18 mF cm^−2^). The observed enhancement in NO_3_RR performance of PB-M compared to PB-2 reveals a correlation with its enhanced ECSAs. To systematically evaluate catalytic efficacy, we conducted electrochemical impedance spectroscopy (EIS) analysis to probe interfacial charge-transfer characteristics (Fig. 3f). PB-Co and PB-Cu demonstrated substantially reduced semicircle diameters relative to PB-2 and PB-Ni, correlating with lower *R*_ct_ values. This charge-transport optimization mechanism effectively enhances the nitrate-to-ammonia conversion kinetics in cobalt-modified systems.

**Fig. 4:**
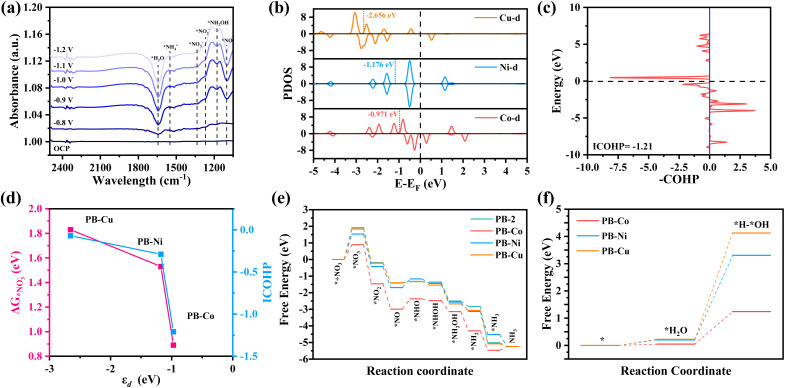
(a) Projected densities of states (PDOSs) of the M (Co, Ni, and Cu) 3d states. (b) PDOS of the M 3d states and NO_3_^−^ 2p states. (c) COHP between Co and NO_3_^−^. (d) The adsorption free energy and ICOHP *versus* d-band center. (e) The calculated Gibbs free energy diagram of NO_3_^−^ to NH_3_ conversion over PB-2 and PB-M. (f) The reaction energies of *H formation over PB-M through H_2_O electrolysis.

*In situ* FT-IR spectrometry was conducted to explore the reaction mechanism of PB-M for the NO_3_RR. As shown in Fig. 4a, the band at ∼1335 cm^−1^ was attributed to the N–O asymmetric stretching vibration of adsorbed NO_3_^−^. The bands located at ∼1100 cm^−1^ and ∼1269 cm^−1^ were assigned to the intermediates *NO and *NO_2_^−^, respectively. The band located at ∼1465 cm^−1^ belonged to N–H bending vibration of *NH_4_^+^, indicating the conversion of NO_3_^−^ to NH_3_ on the catalyst surface under applied potentials. More importantly, we monitored the characteristic peaks of the adsorbed *NH_2_OH species at ∼1178 cm^−1^.^54–56^ According to the above results, the NO_3_RR on PB-Co satisfies the NHO pathway (*NO_3_^−^ → *NO_2_^−^ → *NO → *NHO → *NHOH → *NH_2_OH → *NH_2_ → *NH_3_).

Density functional theory (DFT) calculation was carried out to get deep insight into the great NO_3_RR performance of PB-Co. The electronic configuration of metal d orbitals is an important factor affecting catalytic activity. The projected density of states (PDOS) of PB-Co, PB-Ni, and PB-Cu are plotted in Fig. 4b. It was found that the DOS for spin-up and spin-down states is more asymmetric for PB-Co than for PB-Ni and PB-Cu, indicates that PB-Co has the largest spin density. The calculated d-band centers (*ε*_d_) for PB-Co, PB-Ni, and PB-Cu are −0.971 eV, −1.176 eV, and −2.656 eV, respectively. Previous studies have shown that the more positive the d-band center, the stronger the adsorption energy of NO_3_^−^; therefore, the adsorption of NO_3_^−^ on PB-Co is the strongest.^57^ The interaction between metal d orbitals and NO_3_^−^ 2p orbitals can be explained by the acceptance–donation mechanism:^58,59^ on the one hand, the occupied d orbitals of metal atoms transfer electrons to the empty π* orbitals of NO_3_^−^, on the other hand, the unoccupied d orbitals of metal atoms accept electrons from NO_3_^−^. To further probe the mechanism, the Bader charge and charge density differences were calculated. The Bader charge analysis of PB-M indicates that the Co atom in PB-Co has a more positive charge, which is beneficial to the adsorption of NO_3_^−^ molecules (Fig. S25). Based on the Bader charge and charge density difference of *NO_3_^−^, PB-Co and PB-Ni transferred 0.53 e and 0.56 e to NO_3_^−^, respectively, which are higher than that of PB-Cu (Fig. S26). In order to investigate the orbital interactions between *NO_3_^−^ and metal atoms, we calculated the DOS (Fig. S27) and Crystal Orbital Hamilton Populations (COHP) (Fig. 4c and S28). Analysis of the PDOS between the 2p orbital of *NO_3_^−^ and the metal d orbital of PB-M (M = Co, Ni, Cu) shows that the overlap between the Co d orbital in PB-Co and the *NO_3_^−^ 2p orbital is the largest, which effectively forms strongly chemical bonds of Co–O and then boosts the electrons transfer from Co to NO_3_^−^.^60,61^ The strong adsorption of *NO_3_^−^ on PB-Co is thus validated by COHP, as fewer anti-bonding states are below *E*_F_ when NO_3_^−^ is adsorbed on PB-Co. Furthermore, we calculated the ICOHP values between metal atoms and O atoms, and a more negative ICOHP value for the Co–O bond in PB-Co was observed. Therefore, the adsorption of NO_3_^−^ on PB-Co is stronger than that on PB-Ni and PB-Cu.^62–64^ As shown in Fig. 4d, the closer the d-band center is to the Fermi level, the stronger the adsorption energy for NO_3_^−^ and the d–p orbital hybridization, and the better the NO_3_RR activity. To get deep insight into the superior NO_3_RR performance of PB-Co, the DFT calculation was carried out to investigate the reaction pathways for the NO_3_RR on PB-2 and PB-M. The calculated Gibbs free energy changes along with the related intermediates and reaction pathway are shown in Fig. 4e. The first step involves the adsorption of NO_3_^−^ onto the catalysts, and the stable adsorption of nitrate molecules on catalysts is the key to determining whether the NO_3_RR can occur. The calculated adsorption free energies of PB-2, PB-Co, PB-Ni, and PB-Cu are 1.91, 0.89, 1.53, and 1.83 eV, respectively, indicating that metal atoms can promote the adsorption of NO_3_^−^. There are two typical reaction pathways in the NO_3_RR:^65^ the NOH pathway (*NO_3_ → *NO_2_ → *NO → *NOH → *N → *NH → *NH_2_→ *NH_3_) and the NHO pathway (*NO_3_ → *NO_2_ → *NO → *NHO → *NHOH → *NH_2_OH → *NH_2_ → *NH_3_), and it was found that the NHO pathway is energetically more favorable. The results of theoretical calculation showed that Δ*G*(NO_3_^−^–*NO_3_^−^) was the rate-determining step for both PB-2 and PB-M. The energy barrier of NO_3_^−^ required for PB-Co is 0.89 eV, which is the lowest energy barrier. Therefore, PB-Co exhibits the best NO_3_RR activity and selectivity for NH_3_. NO is an important intermediate in the NO_3_RR, and the degree of activation of NO determines whether subsequent reactions can proceed smoothly. Therefore, we calculated the N–O bond lengths of NO on different catalysts, which were 1.187 Å (PB-Co), 1.175 Å (PB-Ni), and 1.173 Å (PB-Cu), respectively. The maximum elongation of the N–O bond on PB-Co is due to the activation effect of the Co atom on *NO, which is beneficial for the subsequent hydrogenation reaction. To further explore the mechanism of PB-M decomposing water to produce active hydrogen, we also calculated the free energy diagram of water decomposition, as shown in Fig. 4f, Compared with PB-Ni and PB-Cu, H_2_O is more easily adsorbed on PB-Co and more easily decomposed to produce active hydrogen. Therefore, PB-Co exhibits excellent NO_3_RR activity by promoting the adsorption of NO_3_^−^ and H_2_O and producing a large amount of *H.

## Conclusions

In summary, we synthesized three metalized porphyrin organic cages by the post-synthetic metalation of PB-2 and investigated their NO_3_RR performance. Among the investigated PB-2 catalysts with different metal centers, PB-Co exhibited the highest activity, with the trend following the order PB-Co > PB-Ni > PB-Cu. PB-Co exhibited the highest FE_NH3_ of 98.7%, and the corresponding NH_3_ yield rate reached 1089.9 µmol h^−1^ mg_cat_^−1^. Density functional theory (DFT) calculations revealed the mechanism underlying this trend, demonstrating that the proximity of the metal d-band center to the Fermi level (−0.97 eV for PB-Co) governs the d–p orbital hybridization between PB-M and NO_3_^−^. This electronic synergy directly correlates with reduced activation barriers, where the enhanced overlap of Co–NO_3_^–^ orbitals lowers the reaction energy threshold compared to that of PB-Ni and PB-Cu. The experimental and theoretical results also suggest that PB-Co adsorbs H_2_O more easily and produces *H. This work illustrates the intrinsic structure–activity relationship of metalized POCs for the NO_3_RR, which may provide useful guidance for designing efficient electrocatalysts.

## Author contributions

You Wu, Yangpeng Zhang, and Hao Zhao designed the experiments; You Wu synthesized the materials and performed most of the characterization experiments with the help of Yangpeng Zhang, Hao Zhao, Yang Peng and Hailing Ma; You Wu performed the theoretical simulation, interpreted the results and wrote the paper. All authors discussed the results and commented on the manuscript.

## Conflicts of interest

There are no conflicts to declare.

## Supplementary Material

SC-017-D5SC07183F-s001

## Data Availability

The data supporting this article have been included as part of the Supplementary information (SI). Supplementary information: experimental procedures including material synthesis and characterizations; MALDI-TOF, ^1^H NMR, SEM, TEM, EDS, BET, XPS, linear sweep voltammetry curves, faradaic efficiency, Bader charge data, PDOS data and COHP data. See DOI: https://doi.org/10.1039/d5sc07183f.
