# Supplementary material for: Strong d–p orbital hybridization in cobalt porphyrin cages promotes electrochemical nitrate reduction to ammonia
Source: Chem Sci. 2026 Jan 6;17(9):4697–705. doi: 10.1039/d5sc07183f (PMC12797132; doi:10.1039/d5sc07183f)
Supplement: SC-017-D5SC07183F-s001 [file SC-017-D5SC07183F-s001.pdf]

Supplementary Information (SI) for Chemical Science.
This journal is © The Royal Society of Chemistry 2026

Strong d-p Orbital Hybridization in Cobalt Porphyrin Cages Promotes Electrochemical Nitrate Reduction to Ammonia

You Wu,[a,‡] Yangpeng Zhang,[a,‡] Hao Zhao,[a,‡] Yang Peng,[a] Hailing Ma,[a] Fangyuan Kang,[b] Zhonghua Li,[a,*] Yang Liu,[c,*] and Qichun Zhang[b,d,*]

[a] School of Chemistry and Chemical Engineering, Harbin Institute of Technology, Harbin 150001, P. R. China

[b] Department of Materials Science and Engineering, City University of Hong Kong, Hong Kong SAR 999077, P. R. China

[c] School of Computer Science and Technology, Harbin Institute of Technology, Harbin 150001, P. R. China

[d] Department of Chemistry, Center of Super-Diamond and Advanced Films (COSDAF) & Hong Kong Institute of Clean Energy (HKICE), City University of Hong Kong, Hong Kong SAR 999077, P. R. China

*Corresponding author: lizh@hit.edu.cn (Zhonghua Li); liuyang@hit.edu.cn (Yang Liu); qiczhang@cityu.edu.hk (Qichun Zhang)

Materials and Characterization

All the chemicals were commercially available and used without further purification. p-Por-CHO was synthesized based on previous literature. [1]

Synthesis of PB-2.

PB-2 was synthesized according to a literature method with slight modification.[2] p-Por-CHO (200 mg, 274 μmol) and TREN (120.2mg, 822 μmol) were dissolved in dry 1,2-dichlorobenzene (150 mL). Trifluoracetic acid (30 μL) was added to this solution, the reaction was stirred at 80 °C for 5 days under an argon atmosphere. After cooling to room temperature, the reaction mixture was filtered to give dark purple solid which was washed with cold 1,2-dichlorobenzene and methanol. ^{1}H NMR (300 MHZ, $C_2D_2Cl_4$) δ (ppm) 9.24 (s,24H), 8.76-8.65 (m, 48H), 8.44 (s, 24H), 8.04 (brs, 48H), 6.77 (brs, 24H), 4.12 (brs, 24H), 3.65 (brs, 24H), 3.23 (brs, 24H) 2.92 (brs, 24H), -3.60(s, 12H). MALDI-TOF (m/z): $[M]^+$ 5097.3, found: 5097.3.

Synthesis of PB-Co.

PB-2 (102 mg, 20.0 μmol, 1.0 eq.) and $CoCl_2$ (260 mg, 2.0 mmol, 100 eq.) were dissolved in anhydrous THF (100 mL), the reaction mixture was heated at 70 ºC for 24 h under N_2. After cooling to room temperature, the solvent was removed under reduced pressure. Then, CH_3CN (25 mL) was added, and after sonication for 1 min to suspend the product, the mixture was transferred to a conical tube and centrifuged. The supernatant was then decanted. This washing cycle with CH_3CN was repeated eight times. Next, the product was suspended again in CH_3CN (20 mL) and allowed to soak overnight at room temperature. After vacuum filtration and further washing with

MeOH. MALDI-TOF (m/z): $[M]^+$ 5439.3, found:5439.4

Synthesis of PB-Ni

PB-2 (102 mg, 20.0 μmol, 1.0 eq.) and $Ni(NO_3)_2 \cdot 6H_2O$ (581.6 mg, 2.0 mmol, 100 eq.) were dissolved in anhydrous THF (100 mL), the reaction mixture was heated at 70 ºC for 24 h under N_2. After cooling to room temperature, the solvent was removed under reduced pressure. Then, CH_3CN (25 mL) was added, and after sonication for 1 min to suspend the product, the mixture was transferred to a conical tube and centrifuged. The supernatant was then decanted. This washing cycle with CH_3CN was repeated eight times. Next, the product was suspended again in CH_3CN (20 mL) and allowed to soak overnight at room temperature. After vacuum filtration and further washing with MeOH. MALDI-TOF (m/z): $[M]^+$ 5433.3, found:5433.6

Synthesis of PB-Cu

PB-2 (102 mg, 20.0 μmol, 1.0 eq.) and $CuCl_2$ (268.9 mg, 0.6 mmol, 100 eq.) were dissolved in anhydrous THF (100 mL). Subsequently, 2,6-lutidine (74 μL, 0.6 mmol, 30 eq.) was injected, and the reaction mixture was heated at 70 ºC for 24 h under N_2. After cooling to room temperature, the solvent was removed under reduced pressure. Then, CH_3CN (25 mL) was added, and after sonication for 1 min to suspend the product, the mixture was transferred to a conical tube and centrifuged. The supernatant was then decanted. This washing cycle with CH_3CN was repeated eight times. Next, the product was suspended again in CH_3CN (20 mL) and allowed to soak overnight at room temperature. After vacuum filtration and further washing with MeOH. MALDI-TOF (m/z): $[M]^+$ 5469.3, found:5469.5

Electrochemical measurements.

To prepare the working electrode, 5 mg of catalyst power, 1.5 mg of Ketjenblack carbon, and 60 μL of Nafion solution (5 wt%) were dispersed in 940 μL of ethanol and sonicated for 30 min to form a uniform catalyst ink. Then 100 μL catalyst ink was dropcasted on a 1 × 1.5 cm^2 carbon paper and dried at room temperature to achieve an area loading of 0.5 mg cm^{-2}. Electrochemical NO_3RR measurements were performed in a H-type cell with a three-electrode system. Its two compartments were separated by a Nafion 117 membrane. The H-type cell was controlled by a CHI 660E electrochemical workstation, and equipped with an Ag/AgCl electrode and graphite electrode. The electrolyte was Ar-saturated 0.5 M K_2SO_4 containing 0.1 M KNO_3. Linear sweep voltammetry (LSV) was collected at a scan rate of 10 mV s^{-1}. The chronoamperometry tests were conducted at a series of applied potentials in a typical H-type cell that contains 70 mL electrolyte. The catholyte was then collected and analyzed for dissolved NH_3 using UV-Vis.

Determination of the NH_3 concentration.

2 mL of diluted product electrolyte (the product from both two chambers and the outlet gas) was first mixed with 2 mL of 1 M NaOH solution containing 5 wt% salicylic acid and 5wt% sodium citrate. Then 1 mL of 0.05 M NaClO solution and 0.2 mL of 1 wt% sodium nitroferricyanide solution were added subsequently. Then the system was shaken to obtain a uniformly mixed solution. After aging the solution for 2 h, the absorption spectra of the obtained green solution were taken by a UV-Vis spectrophotometer, where the absorbance peak at 655 nm was taken for calculation.

The absorbance was substituted by the standard curve to calculate the ammonia concentration. The standard curve was obtained by a series of different ammonia concentration-absorbance curves prepared with NH_4Cl.

Detection of nitrite-N concentration.

4 g of p-aminobenzenesulfonamide and 0.2 g of N-(1-Naphthyl) ethylenediamine dihydrochloride were dissolved in 10 mL of phosphoric acid. After stirring, it was poured into 50 mL of ultrapure water to obtain Griess reagent. 4 mL of diluted product electrolyte was mixed with 0.1 mL Griess reagent, then shake it for 2 minutes and let it stand for 15 minutes, and its absorption value at 540 nm was measured and recorded. The absorbance was substituted by the standard curve to calculate the nitrite-N concentration. The standard curve was obtained by a series of different nitrate-N concentration-absorbance curves prepared with sodium nitrite.

Isotope labeling experiments.

To identify the nitrogen source for NO_3RR, the reaction was carried out at -0.9 V vs. RHE for 2 h in 0.5 M K_2SO_4 + 0.1 M $K^{14}NO_3$ or 0.5 M K_2SO_4 + 0.1 M $K^{15}NO_3$. At the end of the electrolysis, the catholyte pH was adjusted to 2~4 using diluted 0.1 M HCl. 650 μL of the catholyte was added with 150 μL of D_2O solution containing 20 wt % maleic acid as the internal standard, and then analyzed by ^{1}H-NMR.

Calculation the Faradaic efficiency of NH_3 and NO_2^- yield.

The following equation was used to calculate the rate of ammonia yield:

$$Rate\ of\ NH_3 = \frac{C_{NH_3} \times V}{t \times m}$$

NH_3 Faradaic efficiency (FE_{NH3}) was calculated using the following equation:

$$FE_{NH_3} = \frac{8 \times F \times C_{NH_3} \times V}{Q}$$

where C_{NH3} (μmol L^{-1}) was the ammonia concentration in the catholyte, F was the Faraday constant (96485 C mol^{-1}), V was the volume of electrolyte (70 mL), and Q was the total charge passing the electrode.

The yield of nitrite was Calculated by the following formula:

$$Rate\ of\ NO_2^- = \frac{C_{NO_2^-} \times V}{t \times m}$$

The Faradaic efficiency of nitrite was calculated using the following equation:

$$FE_{NO_2^-} = \frac{F \times C_{NH_3} \times V}{Q}$$

where C (μmol L^{-1}) was the concentration of nitrite in the catholyte, F was the Faraday constant (96485 C mol^{-1}), V was the volume of electrolyte (70 mL), and Q was the total charge passing the electrode.

Computational Details.

All spin polarized DFT calculations were performed by Quantum ESPRESSO code.[3] The Perdew-Burke-Ernzerhof functional within the generalized gradient approximation was used to describe the electron exchange-correlation interactions.[4] The projector-augmented wave (PAW) method was used to describe ion-electron interaction. The Van der Waals interaction is handled by empirical DFT-D3 (BJ) correction.[5,6] A cutoff energy of 60 and 240 Ry was used for the plane-wave basis set. An gamma-centered 1×1×1 k-mesh is uesed for structure optimization. The criteria of energy convergence and force convergence are set to 10^{-6} Ry for the cell and 1×10^{-3} a.u. for each atomic force component. In the DFT calculations, we choose porphyrin ring as the model. The

model was placed in a cubic supercell with lattice parameters of 20 Å × 25 Å × 18 Å to minimize the interaction from periodic images.To avoid calculating the energy of charged NO_3^- directly, gaseous HNO_3 is chosen as a reference instead. The adsorption energy of NO_3^- ($\Delta G*NO_3$) is described as

$$\Delta G_{*NO_3} = -G_{*NO_3} - G_* - G_{HNO_3(g)} + \Delta_{Gcorrect}$$

where G_{*NO3}, $G*$, $G_{HNO3(g)}$, and $G_{H2(g)}$ are the Gibbs free energy of NO_3^- adsorbed on substrates, pure substrates, HNO_3, and H_2 molecules in the gas phase, respectively. $\Delta G_{correct}$ denotes the correction of adsorption energy and is set to 0.392 eV.[7]

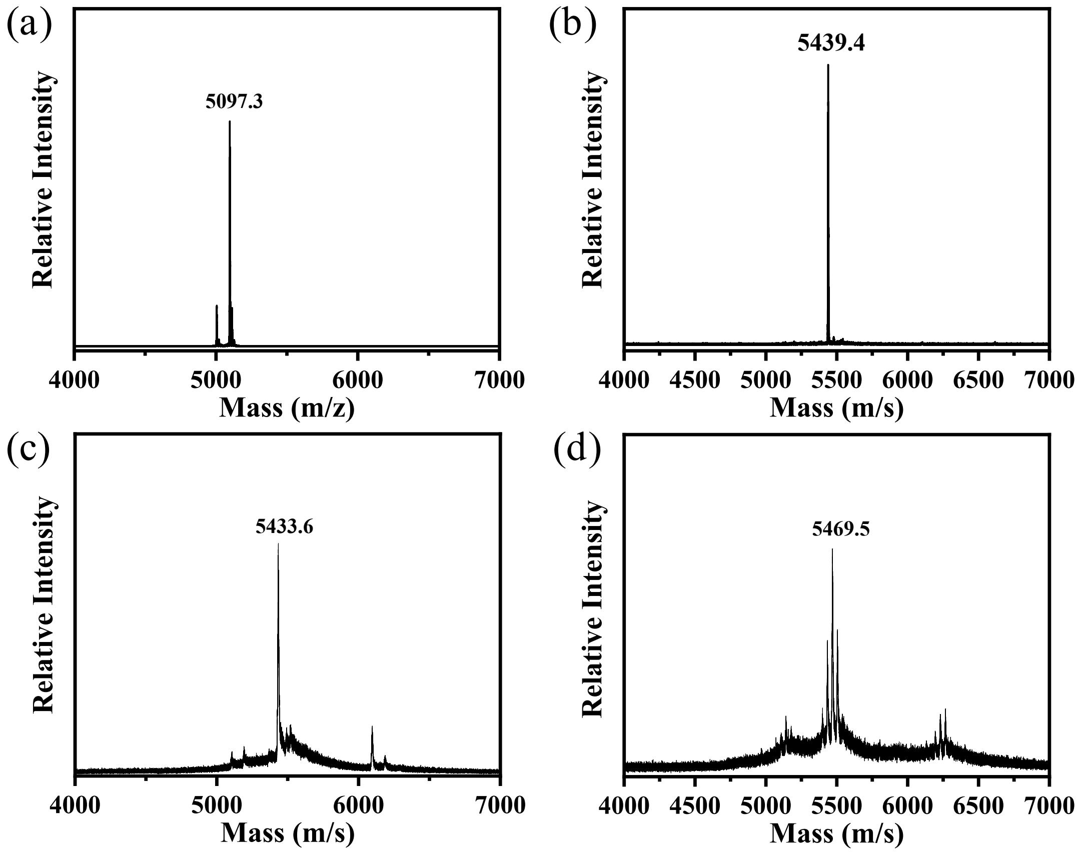

Figure S1. MALDI-TOF mass spectrum of the PB-2 and PB-M (M = Co, Ni, Cu) (Matrix: DCTB). (a) PB-2; (b) PB-Co; (c) PB-Ni; (d) PB-Cu.

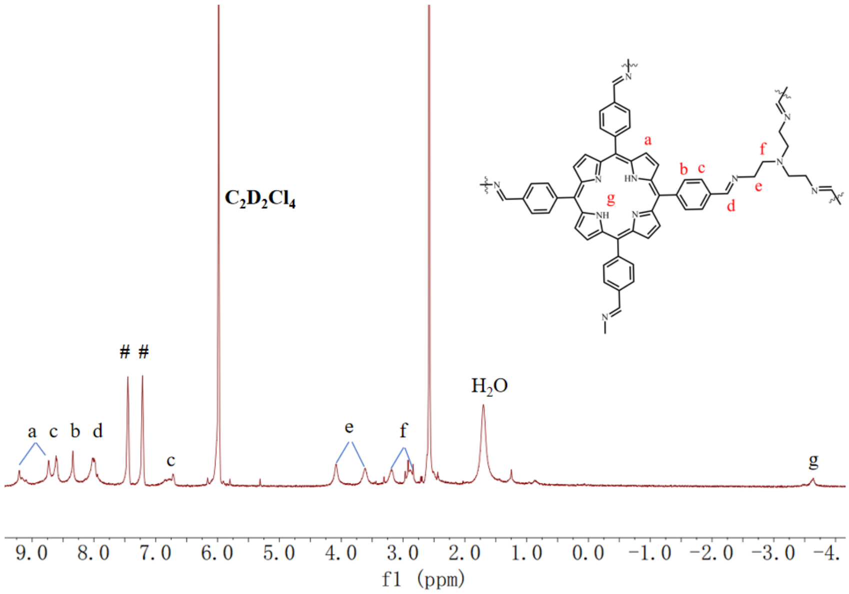

Figure S2. ^{1}H NMR spectrum of the PB-2 ($C_2D_2Cl_4$).

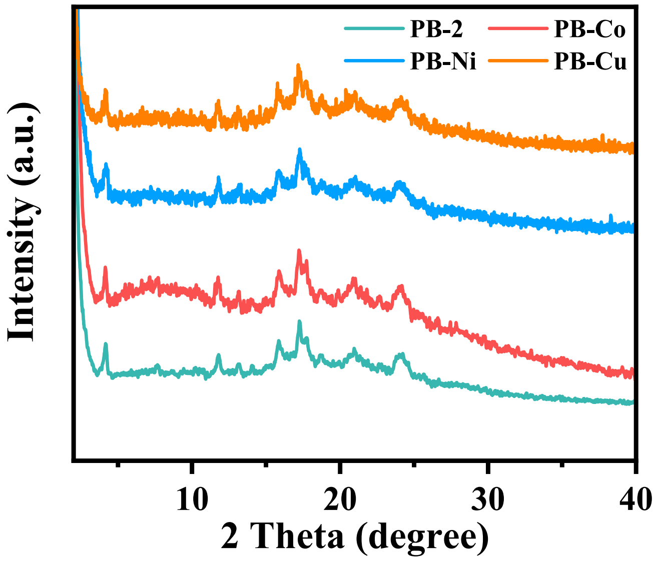

Figure S3. Powder X-ray diffraction of simulated PB-2, synthesized PB-2 and PB-M (M = Co, Ni, Cu)

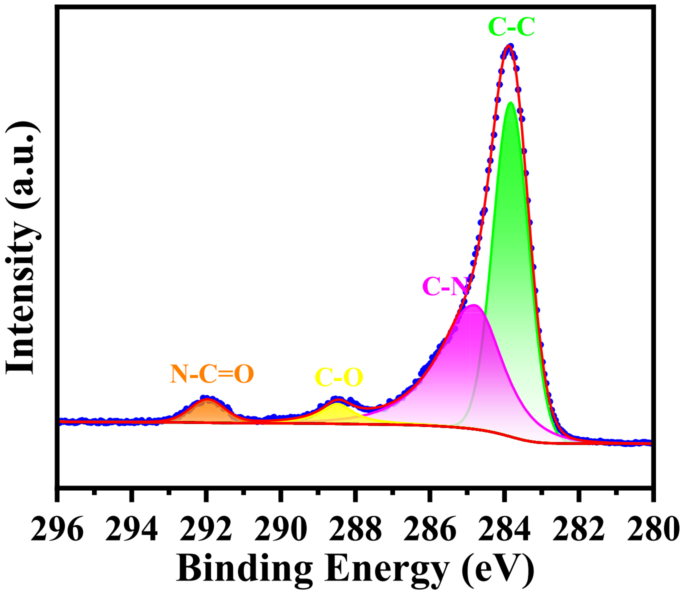

Figure S4. C1s XPS spectra of PB-2.

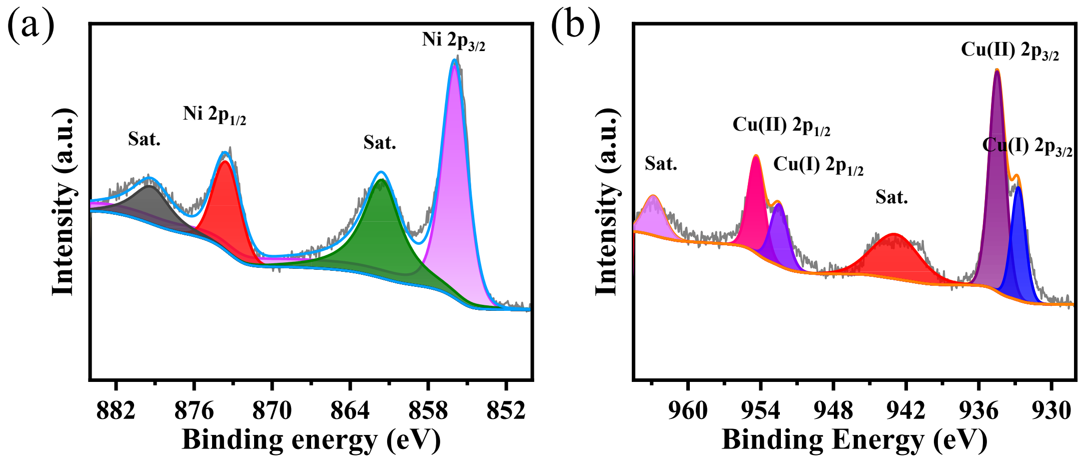

Figure S5. (a) High-resolution Ni 2p XPS spectra of PB-Ni. (b) High-resolution Cu 2p XPS spectra of PB-Cu.

Table S2. The actual content ratio of metal ions (M = Co, Ni, Cu) compared to the theoretical content.

Sample	actual content	theoretical content
PB-Co	6.04 %	6.5 %
PB-Ni	6.27 %	6.5%
PB-Cu	7.18 %	7.0 %

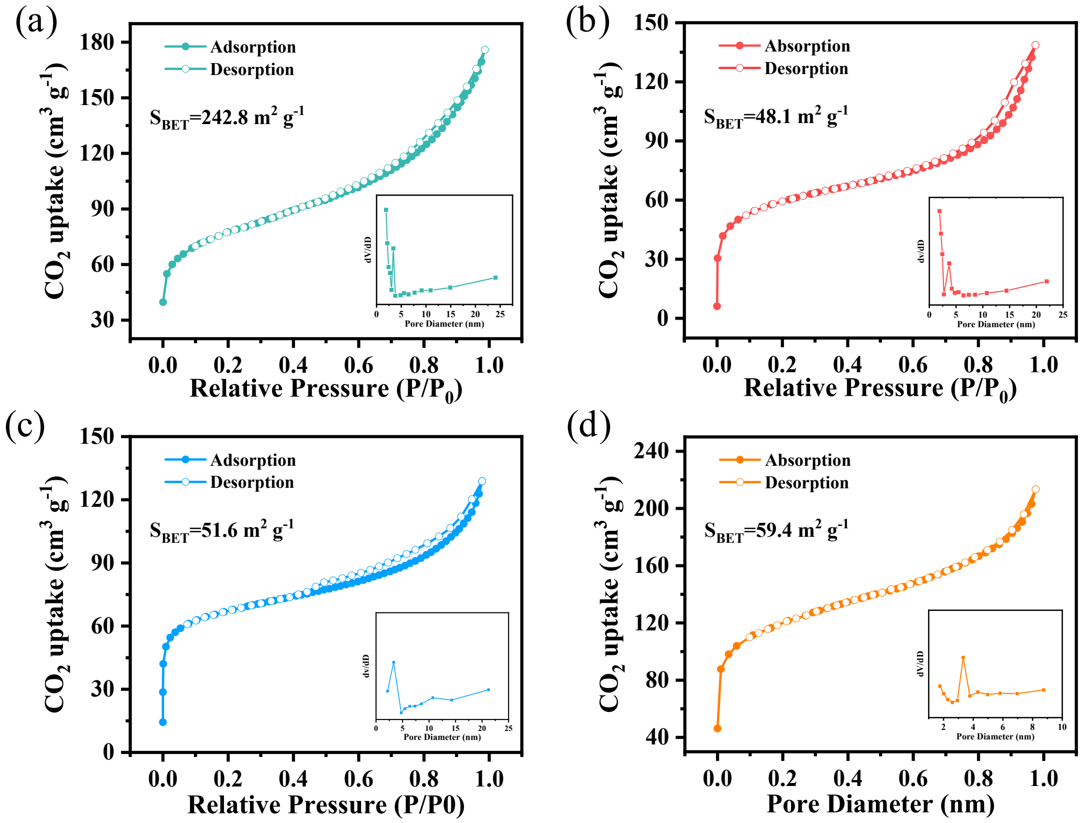

Figure S6. CO_2 adsorption-desorption curves (195 K) and pore width distribution. (a) PB-2; (b) PB-Co; (c) PB-Ni; (d) PB-Cu.

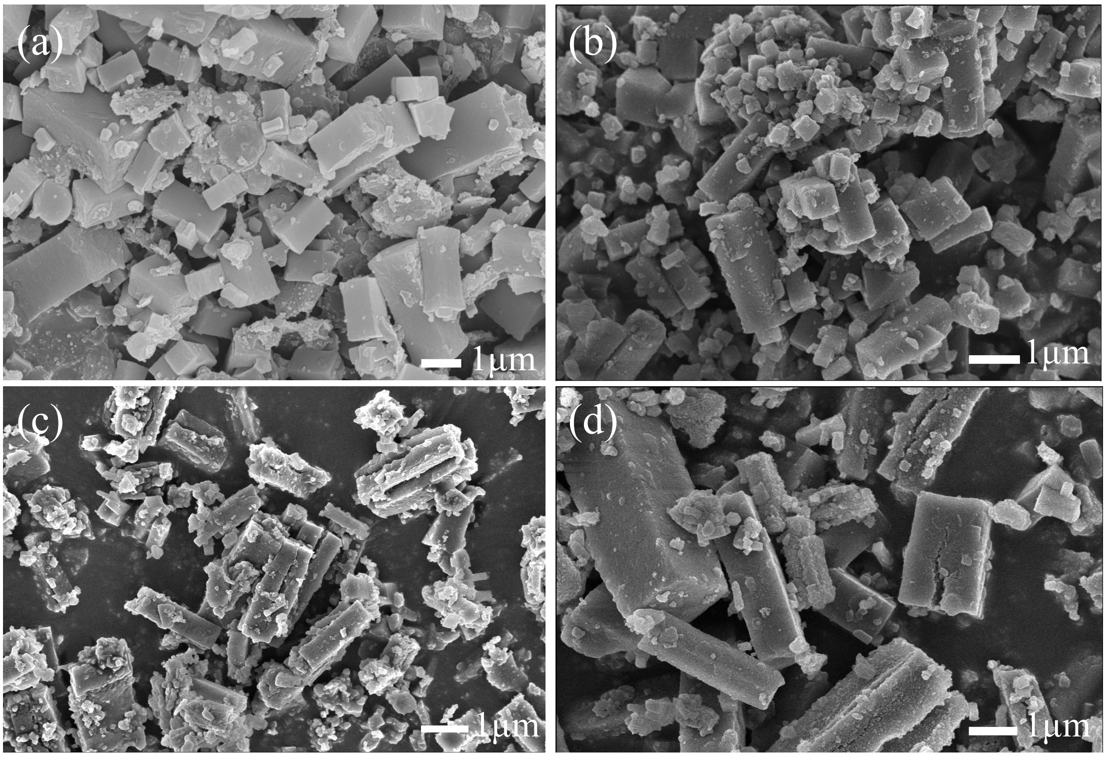

Figure S7. SEM images of (a) PB-2; (b) PB-Co.

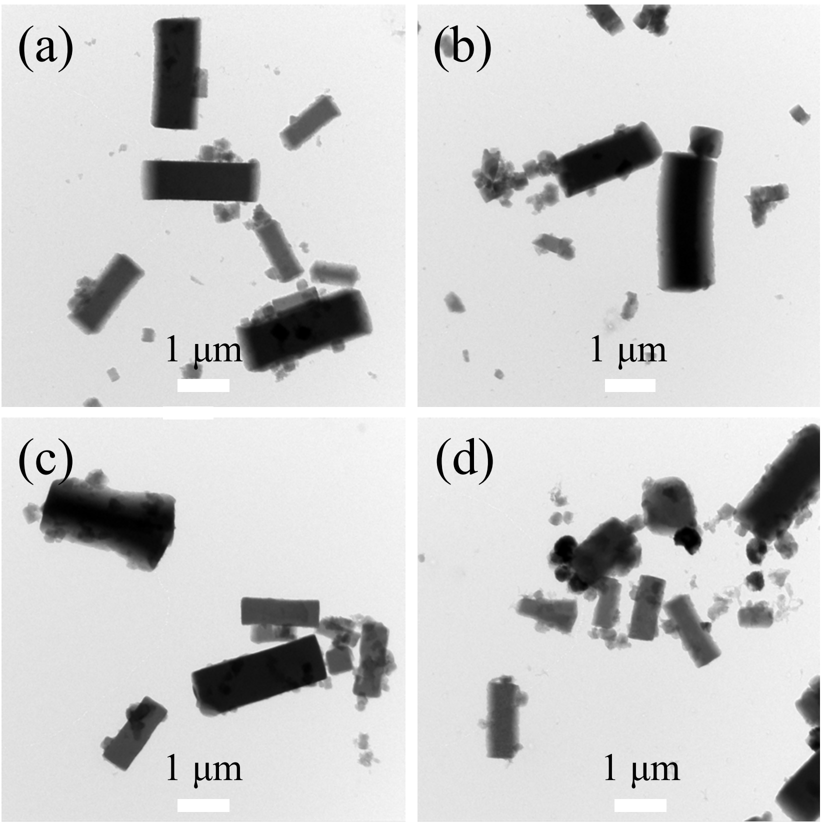

Figure S8. TEM images of (a) PB-2; (b) PB-Co; (c) PB-Ni; (d) PB-Cu.

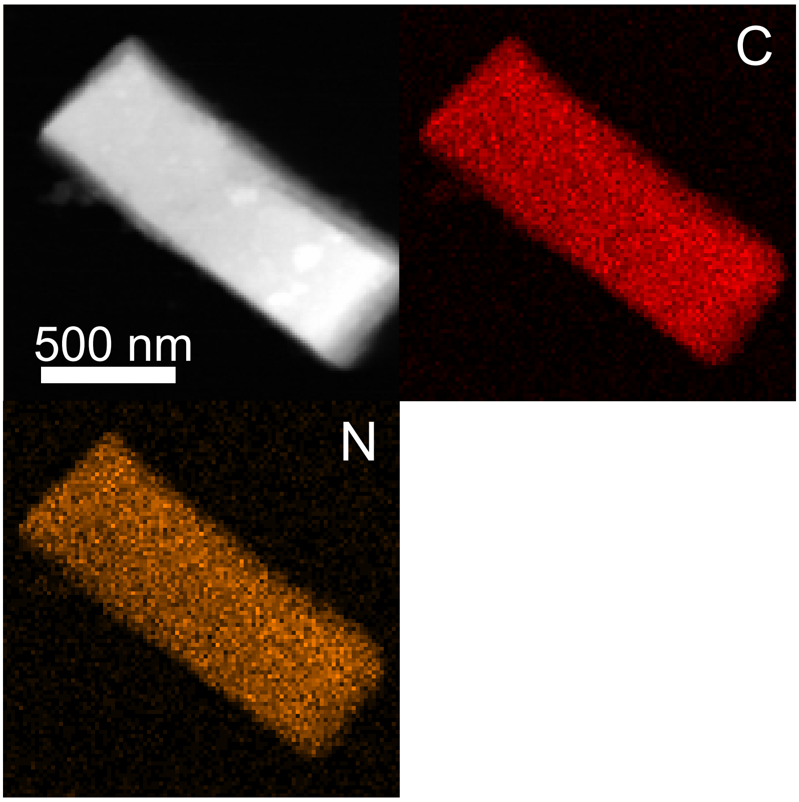

Figure S9. TEM mapping image of PB-2.

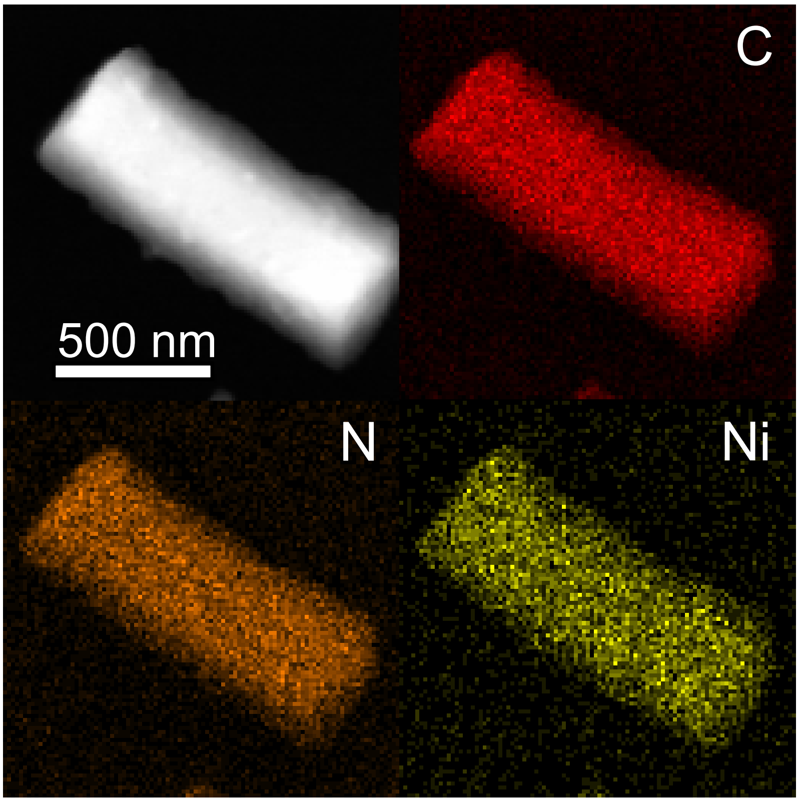

Figure S10. TEM mapping image of PB-Ni.

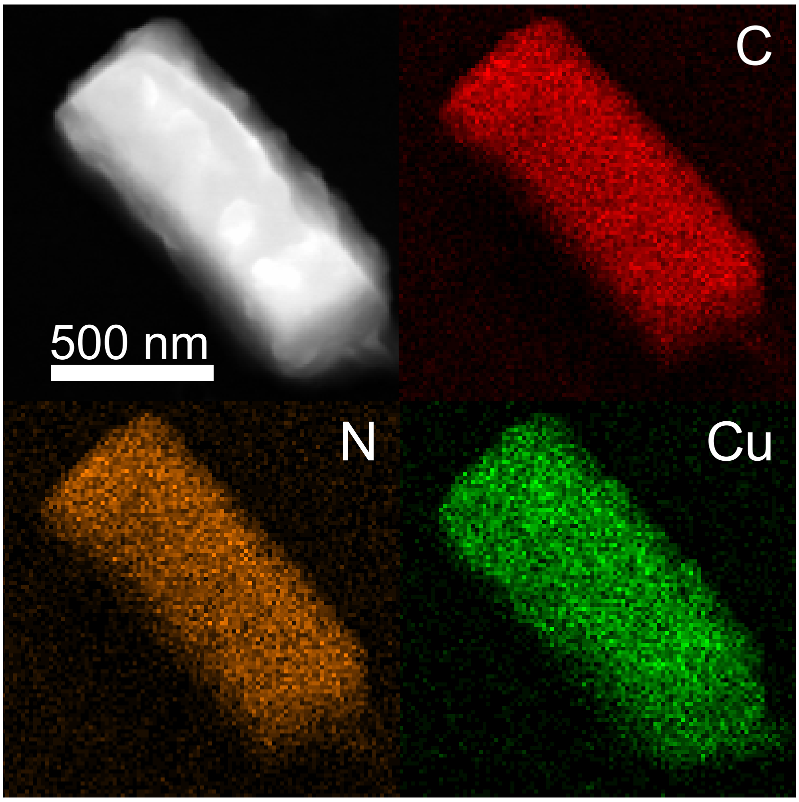

Figure S11. TEM mapping image of PB-Cu.

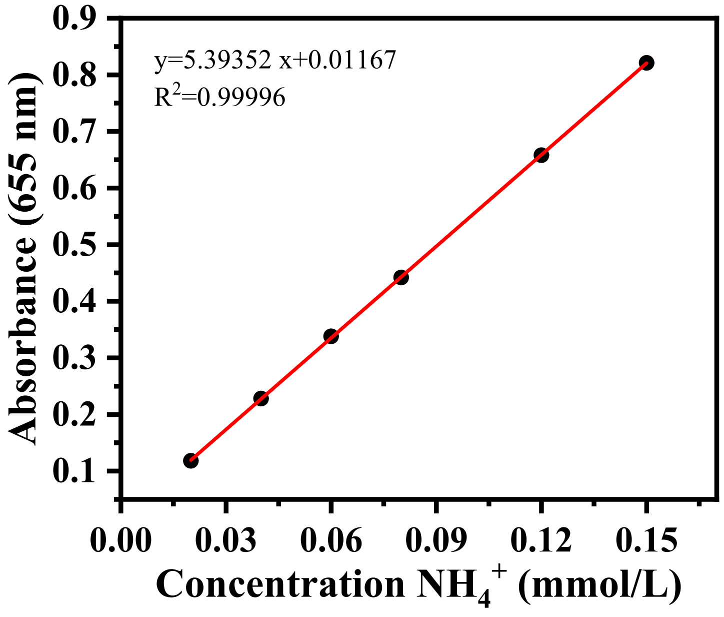

Figure S12. NH_4^+ calibration curve and corresponding best fitting equation.

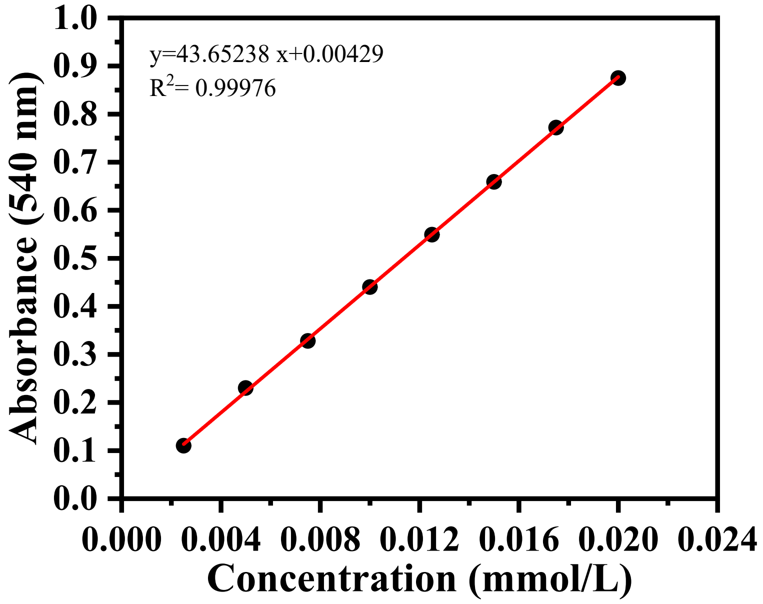

Figure S13. NO_2^- calibration curve and corresponding best fitting equation.

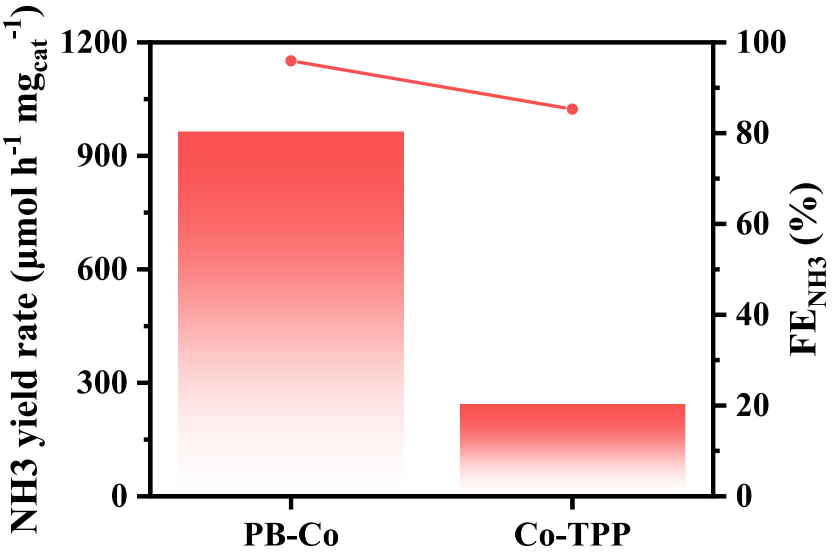

Figure S14. The NH_3 yield rate of PB-Co and Co-TPP.

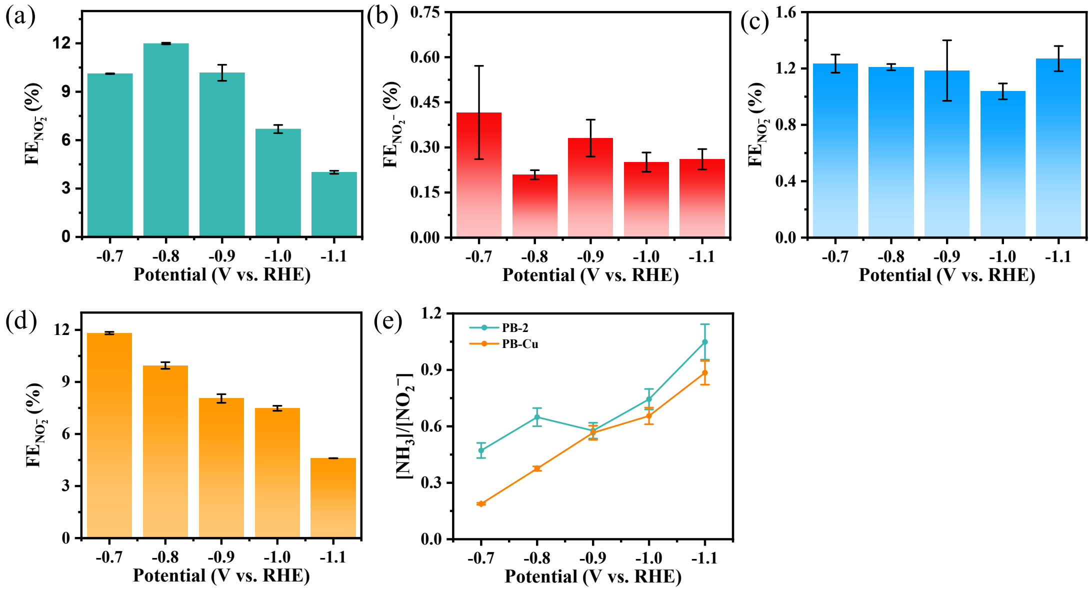

Figure S15. FE of Nitrite for (a) PB-2; (b) PB-Co; (c) PB-Ni; (d) PB-Cu. (e) $[NH_3]/[NO_2^-]$ for PB-2 and PB-Cu at different potential.

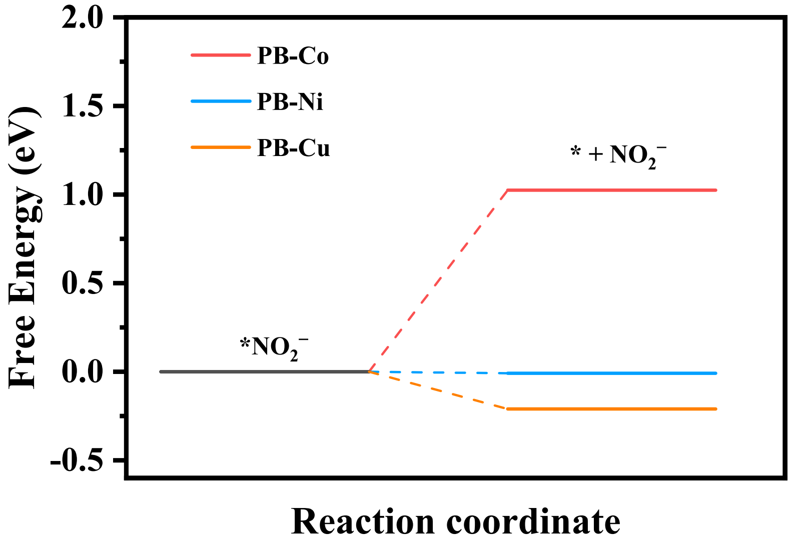

Figure S16. Desorption energy barriers of nitrite on PB-M.

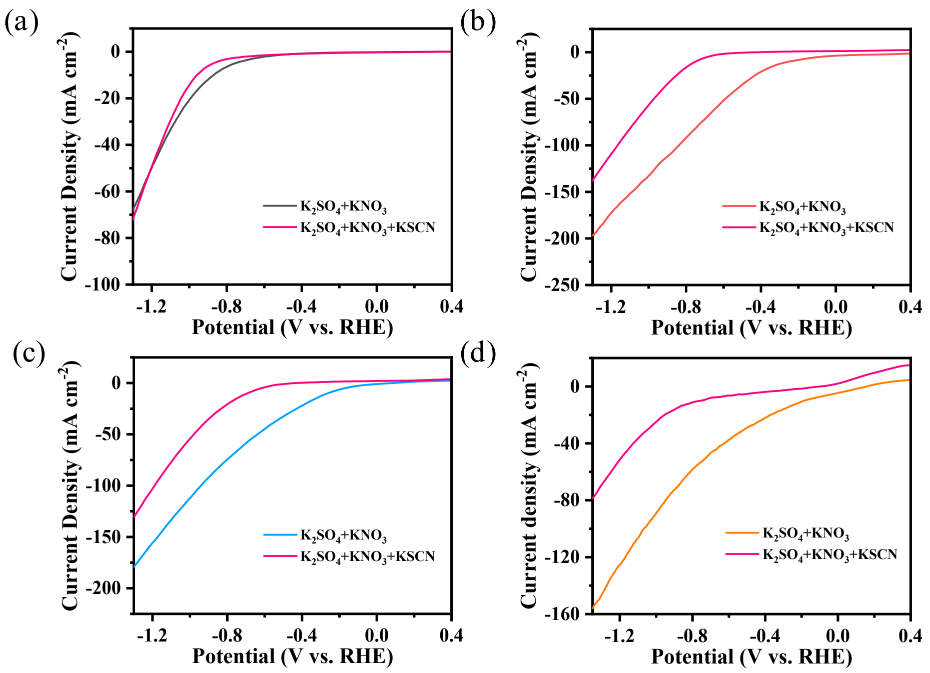

Figure S17. LSV curves of CP and PB-M (M=Ni, Cu) in 0.5 M K_2SO_4 + 0.1 M KNO_3, and 0.5 M K_2SO_4 + 0.1 M KNO_3 + 0.1 M KSCN electrolyte (a) CP; (b) PB-Co; (c) PB-Ni; (d) PB-Cu.

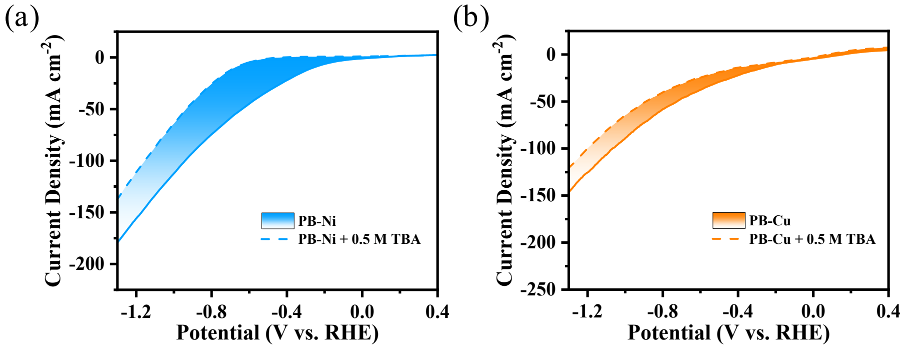

Figure S18. Current density change by adding 0.5 M TBA into KNO_3-containing electrolyte (a) PB-Ni; (b) PB-Cu.

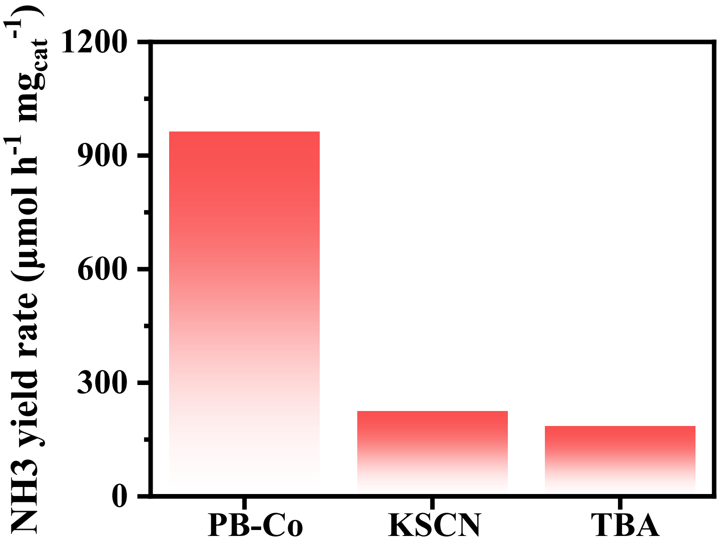

Figure S19. The NH_3 yield rate of PB-Co after the addition of KSCN and TBA into the electrolyte, respectively.

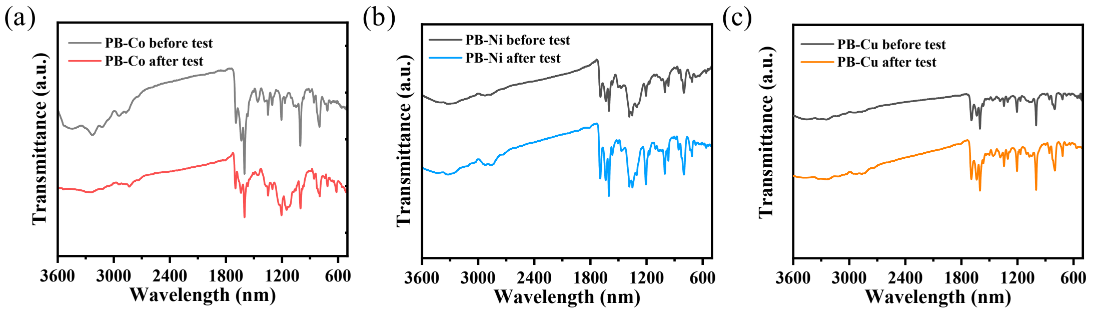

Figure S20. FT-IR spectra of samples before and after the long-term electrolysis. (a) PB-Co; (b) PB-Ni; (c) PB-Cu.

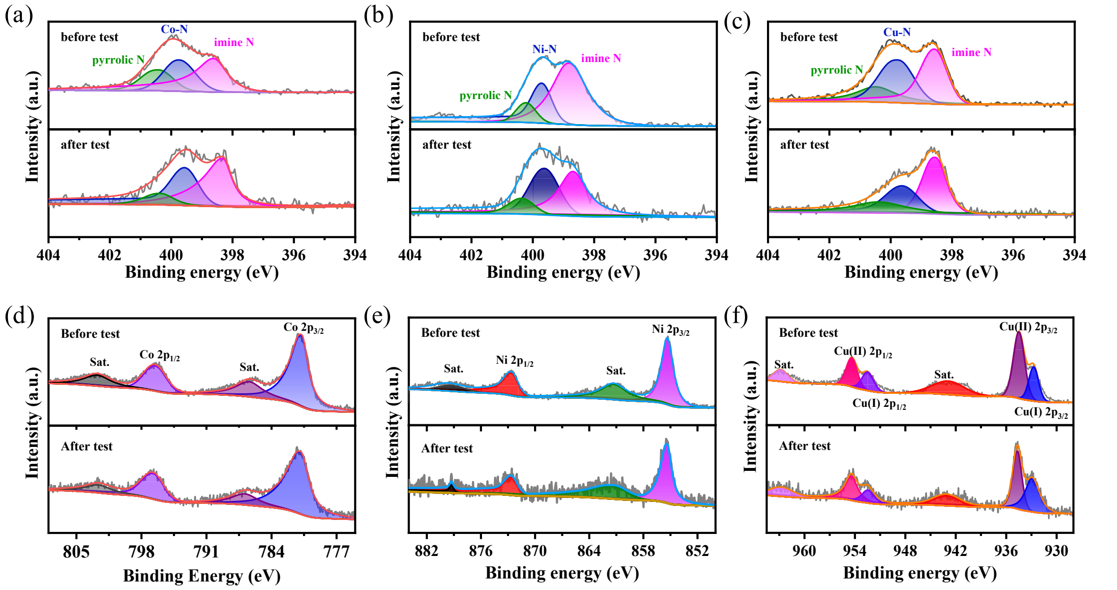

Figure S21. High-resolution N 1s XPS spectra of samples before and after the long-term electrolysis. (a) PB-Co; (b) PB-Ni; (c) PB-Cu. High-resolution M (M = Co, Ni, Cu) 2p XPS spectra of samples before and after the long-term electrolysis. (d) PB-Co; (e) PB-Ni; (c) PB-Cu.

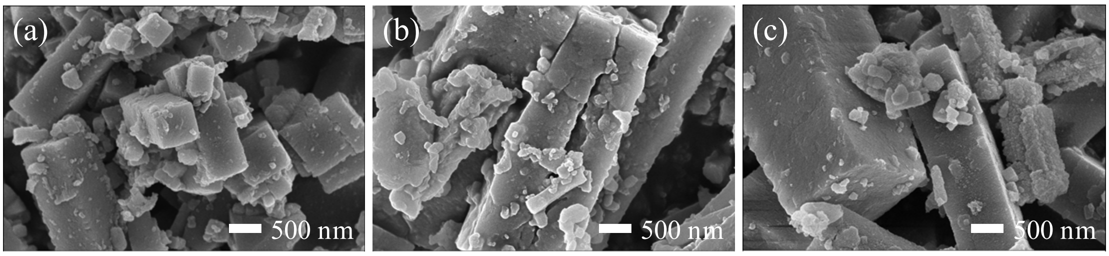

Figure S22. SEM images of samples after the long-term electrolysis. (a) PB-Co; (b) PB-Ni; (c) PB-Cu.

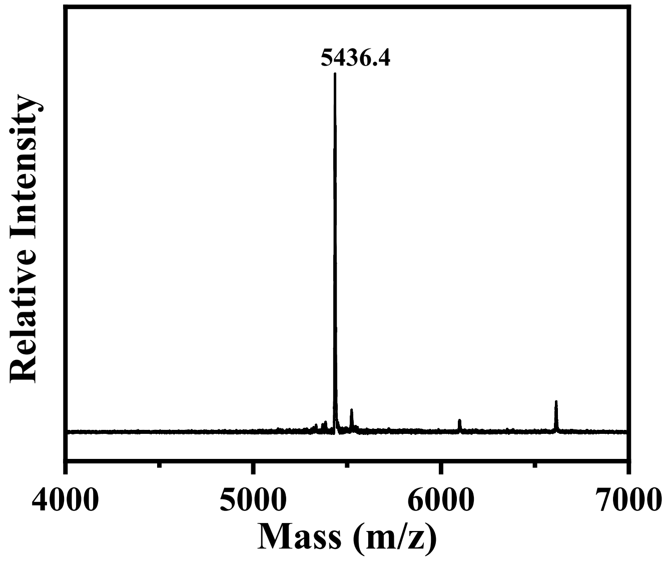

Figure S23. MALDI-TOF mass spectrum of PB-Co after the long-term electrolysis.

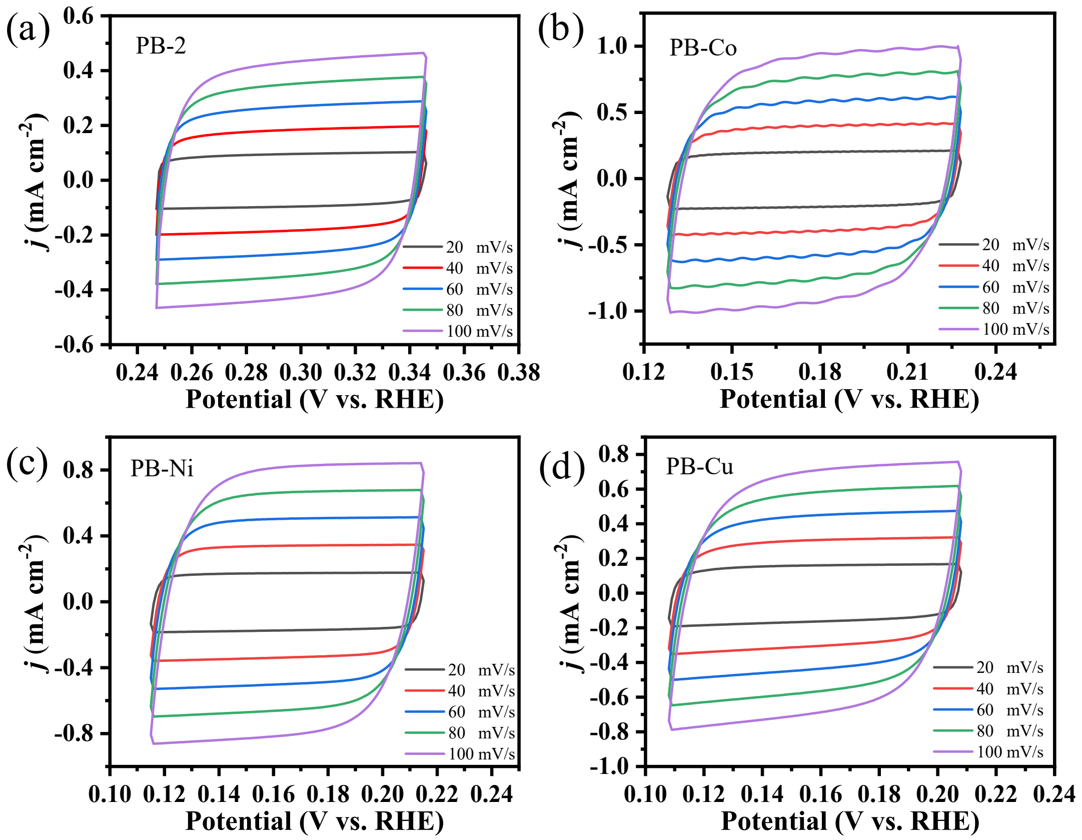

Figure S24. The cyclic voltammetry curves for PB-2 and PB-M in non-Faradic area with scan rates of 20, 40, 60, 80 and 100 mV s^{-1}. (a) PB-2; (b) PB-Co; (c) PB-Ni; (d) PB-Cu.

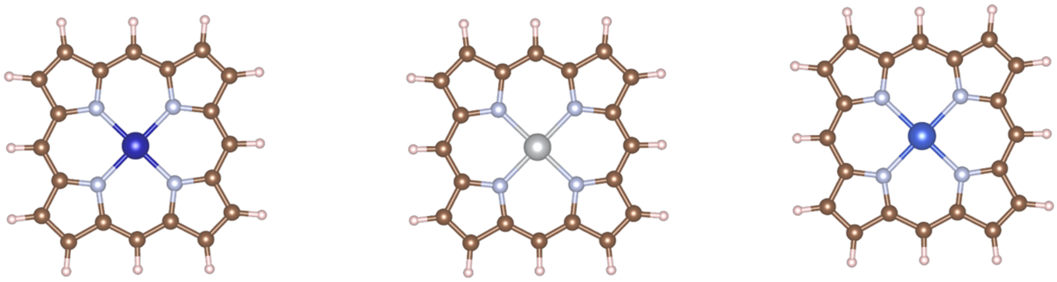

Co:-1.064   **Ni:-0.8818**   **Cu:-0.9434**

Figure S25. Bader charge PB-M (M = Co, Ni, Cu).

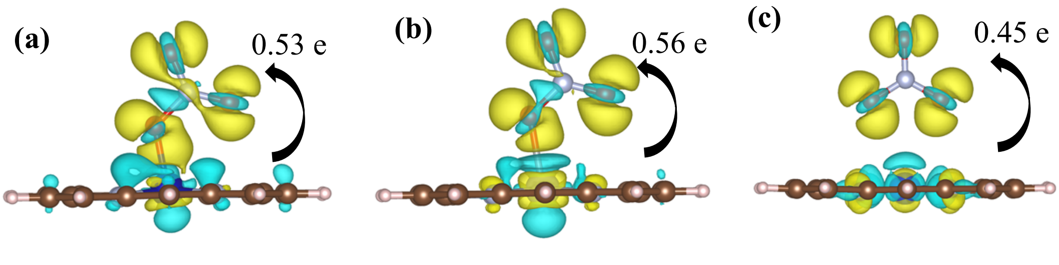

Figure S26. Charge density difference and corresponding charge transfer. (a) PB-Co; (b) PB-Ni; (c) PB-Cu.

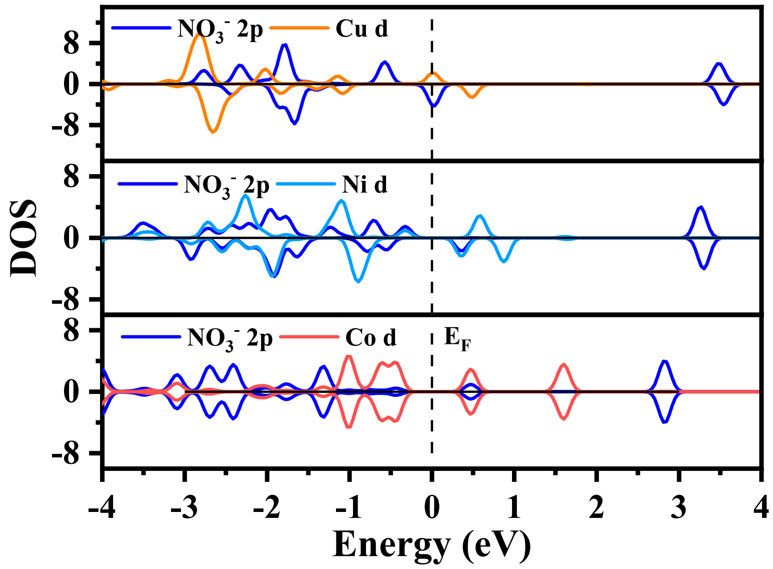

Figure S27. PDOS of the M 3d states and NO_3^- 2p states.

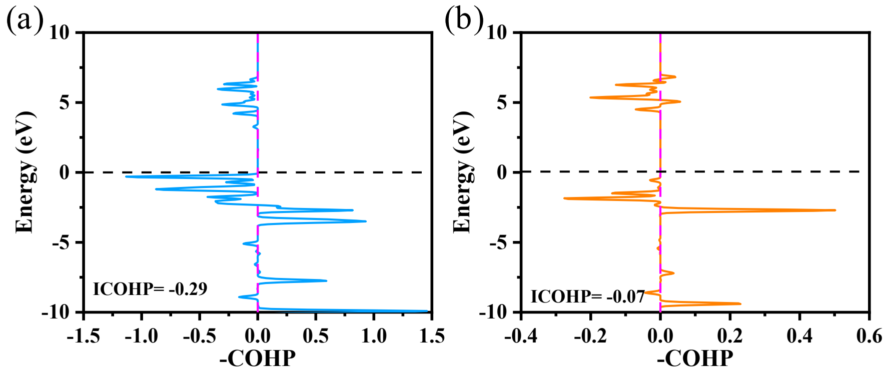

Figure S28. (a) COHP between the Ni and NO_3^-; (b) COHP between the Cu and NO_3^-.

Table S2. Comparison of performance of PB-M with reported catalysts by electrocatalytic nitrate reduction

Catalysts	Electrolyte	Potential (V vs. RHE)	FE (%)	Yield rate (μmol h^{-1} cm^{-2})	Ref
NiPr-TPA-COF	0.5 M K_2SO_4+ 0.1 M KNO_3	-0.75	90	147.1	8
HOF-Cu	0.5 M K_2SO_4+ 0.1 M KNO_3	-1.0	93.8	650	9
$Ni_{1.5}Cu_{1.5}(HITP)_2$	0.1 M Na_2SO_4 + 50 mM $NaNO_3$	−0.9	72.45	130.93	10
Cu_1Co_1HHTP	0.5 M Na_2SO_4 + 0.1 M $NaNO_3$	−0.6	96.4	299.9	11
$Fe(TCNQ)_2$/CF	0.2 M $NaNO_3$ + 0.1 M Na_2SO_4	-1.1	85.2	667.7	12
CoQPyPhenI/CNT	0.1 M K_2SO_4+ 0.1 M KNO_3	-0.6	94.29	533.8	13
Cu–Fe–N–C	1 M KOH + 0.1 M KNO_3	-0.8	95.08	1220	14
Mn–O–C	0.1 M K_2SO_4 + 1000 ppm KNO_3	-0.5	89.0	86.8	15
COF-366-Fe	0.5 M K_2SO_4+ 0.1 M KNO_3	-1.7 vs. SCE	85.4	169.52	16
CuPOR-COF	1 M KOH + 1 M KNO_3	-1.7	86	352.9	17
NiTP-CoTAPP MCOF	0.5 M KNO_3	-0.8	85.6	64.08	18

Ni-TAPP-Cu	0.05 M K_2SO_4 + 0.5 M KNO_3	−1.3	86.13	273.7	19
CoFe-cMOF	1 M Na_2SO_4 + 0.5 M KNO_3	−0.7	94.3	829.4	20
TpBpy−Cu−F	0.5 M Na_2SO_4 + 0.5 M $NaNO_3$	−0.746	92.7	876.5	21
BECOF/Pd CuCu	0.05 M H_2SO_4 + 0.1 M KNO_3	-1.3	91.0	2202	22
Ru-Tta-Dfp	1 M KOH + 0.1 M KNO_3	-0.4	93.93	68.2	23
NiPc-CZDM-COF	0.5 M K_2SO_4 + 0.1 M KNO_3	−1.0	99.8	1147.1	24
ImPy-COF-Mn	NO_3^-(2mg mL^{-1}) + 1.0 M KOH + 0.5 M KF	-0.7	95.64	963.5	25
Ni-MOF-Ru	1 M KOH + 1 M KNO_3	-0.6	91.5	1310	26
Cu_{12}-NND-H	0.1 M $KHCO_3$ + 0.05 M KNO_3	-0.7	98.7	619.4	27
PCN-Cu-DAC	1 M KOH + 1 M KNO_3	−0.78	97.5	6000	28
Fe_2Co-MOF	0.05 M H_2SO_4 + 50 g L^{-1} KNO_3	−1.1	90.55	101.9	29
**PB-Co**	**0.5 M K_2SO_4+ 0.1 M KNO_3**	**-0.9**	**95.8**	**497.8**	**This work**
**PB-Ni**	**0.5 M K_2SO_4+ 0.1 M KNO_3**	**-0.7**	**78.4**	**251.1**	**This work**
**PB-Cu**	**0.5 M K_2SO_4+ 0.1**	**-1.0**	**78.5**	**234.3**	**This work**

	**M KNO_3**

Reference:

1 T. Wang, L. Guo, H. Pei, S. Chen, R. Li, J. Zhang and T. Peng, *Small*, 2021, **17**, 2102957.

2 S. Hong, Md. R. Rohman, J. Jia, Y. Kim, D. Moon, Y. Kim, Y. H. Ko, E. Lee and K. Kim, *Angew. Chem. Int. Ed.*, 2015, **54**, 13241–13244.

3 P. Giannozzi, S. Baroni, N. Bonini, M. Calandra, R. Car, C. Cavazzoni, D. Ceresoli, G. L. Chiarotti, M. Cococcioni, I. Dabo, A. Dal Corso, S. De Gironcoli, S. Fabris, G. Fratesi, R. Gebauer, U. Gerstmann, C. Gougoussis, A. Kokalj, M. Lazzeri, L. Martin-Samos, N. Marzari, F. Mauri, R. Mazzarello, S. Paolini, A. Pasquarello, L. Paulatto, C. Sbraccia, S. Scandolo, G. Sclauzero, A. P. Seitsonen, A. Smogunov, P. Umari and R. M. Wentzcovitch, *J. Phys.: Condens. Matter*, 2009, **21**, 395502.

4 J. P. Perdew, K. Burke and M. Ernzerhof, *Phys. Rev. Lett.*, 1996, **77**, 3865–3868.

5 S. Grimme, S. Ehrlich and L. Goerigk, *J. Comput. Chem.*, 2011, **32**, 1456–1465.

6 S. Grimme, J. Antony, S. Ehrlich and H. Krieg, *J. Chem. Phys.*, 2010, **132**, 154104.

7 H. Niu, Z. Zhang, X. Wang, X. Wan, C. Shao and Y. Guo, *Adv. Funct. Mater.*, 2021, **31**, 2008533.

8 F. Lv, M. Sun, Y. Hu, J. Xu, W. Huang, N. Han, B. Huang and Y. Li, *Energy Environ. Sci.*, 2023, **16**, 201–209.

9 P. Zhai, C. Wang, Y. Li, D. Jin, B. Shang, Y. Chang, W. Liu, J. Gao and J. Hou, *Nano Lett.*, 2024, **24**, 8687–8695.

10 J. Yan, J. Li, P. Liu, H. Huang and W. Song, Green Chem., 2023, **25**, 8645–8651.

11 P. Liu, J. Yan, H. Huang and W. Song, *Chem. Eng. J.*, 2023, 466, 143134.

12 N. Mukherjee, A. Adalder, N. Barman, R. Thapa, R. Urkude, B. Ghosh and U. K. Ghorai, *J. Mater. Chem. A*, 2024, **12**, 3352–3361.

13 L. Sun, C. Dai, T. Wang, X. Jin, Z. J. Xu and X. Wang, *Angew. Chem. Int. Ed.*, 2024, **63**, e202320027.

14 X. Zhang, X. Liu, Z.-F. Huang, L. Gan, S. Zhang, R. Jia, M. Ajmal, L. Pan, C. Shi, X. Zhang, G. Yang and J.-J. Zou, Energy Environ. Sci., 2024, **17**, 6717–6727.

15 S. Zhang, Y. Zha, Y. Ye, K. Li, Y. Lin, L. Zheng, G. Wang, Y. Zhang, H. Yin, T. Shi and H. Zhang, *Nano-Micro Lett.*, 2024, **16**, 9.

16 H. Hu, R. Miao, F. Yang, F. Duan, H. Zhu, Y. Hu, M. Du and S. Lu, *Adv. Energy Mater.*, 2024, **14**, 2302608.

17 S. I. G. P. Mohamed, S. Namvar, T. Zhang, H. Shahbazi, Z. Jiang, A. M. Rappe, A. Salehi-Khojin and S. Nejati, *Adv. Mater.*, 2024, **36**, 2309302.

18 Z. Zhang, M. Wang, H. Xing, X. Zhou, L. Gao, S. Chen, Y. Chen, H. Xu, W. Li, S. Yuan, C. Li, Z. Jin and J. Zuo, *Angew. Chem. Int. Ed.*, 2025, **64**, e202505580.

19 S.-W. Ke, Y. Lv, Y. Gu, J. Su, L. Tang, L. Fang, S. Yuan, J. Ma, M. Ding and J.-L. Zuo, *JACS Au*, 2025, **5**, 2523–2532.

20 S. Tian, R. Wu, H. Liu, C. Yan, Z. Qi, P. Song, W. Chen, L. Song, Z. Wang and C. Lv, *Angew. Chem. Int. Ed.*, 2025, **64**, e202510665.

21 Y. Zhu, H. Duan, C. G. Gruber, W. Qu, H. Zhang, Z. Wang, J. Zhong, X. Zhang, L. Han, D. Cheng, D. D. Medina, E. Cortés and D. Zhang, *Angew. Chem. Int. Ed.*, 2025, **64**, e202421821.

22 Q. Cheng, S. Liu, Y. He, M. Wang, H. Ji, Y. Huan, T. Qian, C. Yan and J. Lu, *Nat. Commun.*, 2025, **16**, 3717.

23 A. Chaturvedi, S. Gaber, S. Kaur, K. C. Ranjeesh, T. C. Nagaiah and D. Shetty, *ACS Energy Lett.*, 2024, **9**, 2484–2491.

24 M. Li, B. Han, L. Gong, Y. Jin, M. Wang, X. Ding, D. Qi and J. Jiang, *Chin. Chem. Lett.*, 2024, 110590.

25 X. Li, S. Xia, S. Yang, X. Yang, S. Zheng, X. Xu, Y. Wang, Q. Xu and Z. Jiang, *Angew. Chem. Int. Ed.*, 2025, **64**, e202507479.

26 Y. Yao, X. Wei, H. Zhou, K. Wei, B. Kui, F. Wu, L. Chen, W. Wang, F. Dai, P. Gao, N. Wang and W. Ye, *ACS Catal.*, 2024, **14**, 16205–16213.

27 Q. Li, L. Luo, X. Guo, R. Wang, J. Liu, W. Fan, Z. Feng and F. Zhang, *J. Am. Chem. Soc.*, 2025, 147, 1884–1892.

28 Q. Li, Y. Li, B. Xu, J. Yang and Y. Wang, *Angew. Chem. Int. Ed.*, 2025, **64**, e202510139.

29 Y. Lv, S. Ke, Y. Gu, B. Tian, L. Tang, P. Ran, Y. Zhao, J. Ma, J. Zuo and M. Ding, *Angew. Chem. Int. Ed.*, 2023, **135**, e202305246.
